# A review of volume‐area scaling of glaciers

**DOI:** 10.1002/2014RG000470

**Published:** 2015-02-24

**Authors:** David B. Bahr, W. Tad Pfeffer, Georg Kaser

**Affiliations:** ^1^ Department of Physics and AstronomyRegis CollegeDenverColoradoUSA; ^2^ Institute of Arctic and Alpine ResearchUniversity of Colorado at BoulderBoulderColoradoUSA; ^3^ Institute of Meteorology and GeophysicsUniversity of InnsbruckInnsbruckAustria

**Keywords:** glaciers, glacier mass balance, power law scaling

## Abstract

Volume‐area power law scaling, one of a set of analytical scaling techniques based on principals of dimensional analysis, has become an increasingly important and widely used method for estimating the future response of the world's glaciers and ice caps to environmental change. Over 60 papers since 1988 have been published in the glaciological and environmental change literature containing applications of volume‐area scaling, mostly for the purpose of estimating total global glacier and ice cap volume and modeling future contributions to sea level rise from glaciers and ice caps. The application of the theory is not entirely straightforward, however, and many of the recently published results contain analyses that are in conflict with the theory as originally described by Bahr et al. (1997). In this review we describe the general theory of scaling for glaciers in full three‐dimensional detail without simplifications, including an improved derivation of both the volume‐area scaling exponent *γ* and a new derivation of the multiplicative scaling parameter *c*. We discuss some common misconceptions of the theory, presenting examples of both appropriate and inappropriate applications. We also discuss potential future developments in power law scaling beyond its present uses, the relationship between power law scaling and other modeling approaches, and some of the advantages and limitations of scaling techniques.

## Key Points

- Glacier scaling relationships are derived from dimensional analysis
- No simplifying assumptions are necessary
- Common misconceptions are discussed

## 1 Introduction

Power law representations of natural phenomena, and the application of dimensional analysis and rescaling operations, have been conspicuously successful in the analysis of complex systems in physics, biology, and various natural sciences for more than a century. Methods of partial quantitative analysis, which seek to characterize or place constraints on the solution of problems that are either unsolved or not fully formulated, have been pursued since the time of Newton. Related concepts and practices identified not only by Newton but also by Maxwell, Fourier, and others were applied to problems in mechanics, electrodynamics, fluid mechanics, and heat transfer. These eventually became codified in principles now known variously as geometric, kinematic, and dynamic similitude, dimensional analysis, and in many cases have led to well‐known power law structures. The widespread occurrence of power law‐like relationships in nature has been noted in fields as widespread as biology [*Huxley and Teissier*, 1936; *Gayon*, 2000], sociology [*Gerchak*, 1984] economics [*Andriani and McKelvey*, 2007], lexicographic analysis [*Alexopoulou et al*., 2009; *Schmidt and Housen*, 1995], and most relevant to the present purpose, geomorphology and geologic processes [*Horton*, 1945; *Strahler*, 1957; *Dodds and Rothman*, 2000]. The ubiquity of power law‐type structure in natural processes has been explained in terms of statistical theory [e.g., *Pietronero et al*., 2001] and is understood to be shaped by specific aspects of a given application but ultimately determined by very broad principals.

However, not all purported power laws are supported by rigorous statistical tests of the data [*Clauset et al*., 2009], and much greater confidence can be placed in an observed power law if it is also derived from underlying math and physics. This can be exceptionally difficult in many disciplines (literary theory, for example), but for a few fields, rigorous derivations have been tremendously successful and help explain very complex phenomena in exceedingly simple terms. Statistical mechanics, for example, has a long history of using physics to derive observed power law scaling behaviors near phase transitions and critical points [e.g., *Ma*, 1976; *Stanley*, 1999], and scaling in statistical physics has been the source of a Nobel Prize (Kenneth G. Wilson in 1982). However, as reasonably expected, the scaling relationships in statistical physics appear simple but are subtle. The same is true in glaciology.

The analysis and interpretation of power law‐type phenomena require tools that apply to the general characteristics of a process (e.g., relevant independent variables) rather than to first‐principles computation (e.g., calculation of forces from conservation of momentum). The earliest analyses of this type were all based on the concept of commensurability, or dimensional homogeneity. First identified by Newton, commensurability recognizes the fact that any valid algebraic expression must be composed of variables quantified by units of measurement that appear in the same ratio in all algebraic terms of the expression. Without this condition, the relative magnitude of terms in the expression could possibly be altered simply by changing some unit of measurement (e.g., lengths measured in feet versus meters, or time measured in seconds versus years). Underlying the principle of similitude is the seemingly self‐evident but powerful assumption that quantification of phenomena (or indeed mathematical expressions independent of any physical entity) must not be altered by a change of units chosen to measure variables. This and other semi‐quantitative principles applied by early investigators are today referred to collectively as Dimensional Analysis, an essential condition of which was codified as the “Buckingham Pi Theorem” in the early twentieth century [*Buckingham*, 1914]. While the Pi Theorem is the best known and most widely applied result of the theory of dimensional analysis, other valuable supplementary principles, much less well known, are also available, such as the principle of Orientational Similitude [*Siano*, 1985a, 1985b].

Among the best known results of dimensional analysis are the dimensionless groups defined in the nineteenth and twentieth centuries in mass and heat transfer, including such widely used terms as the Reynolds, Nusselt, Prandtl, and Péclet numbers. Like all dimensionless quantities, these describe the *average* or *characteristic* behavior of a material or system, assigning order‐of‐magnitude values to relationships such as (in the case of the Reynolds number) the relative importance of momentum versus viscosity or (in the case of the Péclet number) the relative importance of diffusion versus advection. In no case does the result of dimensional analysis provide a specific result applicable to a specific state of the system in question; just as the terms in the dimensionless quantities combine their *approximate* values to form an *approximate* dimensionless ratio, the application of the result is approximate in nature and cannot be used to extract a precise result for any given set of conditions.

In geomorphology, power law relationships, dimensional analysis, and the principle of similarity have become foundational principles that introduce an orderly structure for understanding the structure of topography [*Meybeck*, 1995], hydrocarbon deposits [*Hewitt*, 1986] and the geometric structure of river drainage basins [*Horton*, 1945; *Turcotte*, 1997; *Dodds and Rothman*, 2000]. The role of power law scaling in describing these structures shares many aspects with the description of glacier characteristics here. The application of power law scaling to glaciers shares a common theoretical basis with the analysis of other landforms, as well as with results in fluid mechanics; scaling is neither an untested empirical result nor a new and unproven concept.

This review presents an overview of power law methods used in glaciology. These methods, variously referred to as “macroscopic” methods [*Harrison*, 2013], “power law scaling,” “volume‐area scaling,” or simply “scaling methods,” are widely used, have a well‐established theoretical foundation, and yet suffer from a lack of a common basis for understanding their formulation and validity. Our objective in this summary is to present a complete derivation of scaling methods in glaciology, starting from first principals, and to draw parallels between these techniques and much older and more familiar results such as those discussed above.

A traditional approach to scaling and dimensional analysis has been used in glaciology since at least the 1970's to facilitate various shallow‐ice approximations [e.g., *Fowler and Larson*, 1978] and to derive general ice sheet flow solutions as extensively reviewed and expanded upon in *Baral et al*. [2001]. In this customary approach, the dimensional analysis is used to derive a set of dimensionless (scale invariant) quantities that must remain unchanged as a glacier adjusts its size. For example, a glacier that has twice the surface area should have the same dimensionless quantities as the original‐sized glacier. This scale invariance becomes the source of power law behaviors because size is irrelevant—large and small glaciers are simply rescaled versions of each other. In glacier dynamics, the scale invariance is only possible if most of the dimensionless quantities are declared negligible so that they are always near zero and therefore nearly the same for all glaciers. For example, the aspect ratio of thickness to length must remain the same (very small) at all times for all glaciers. This leads to the so‐called “shallow‐ice approximation.”

**Figure 1:**
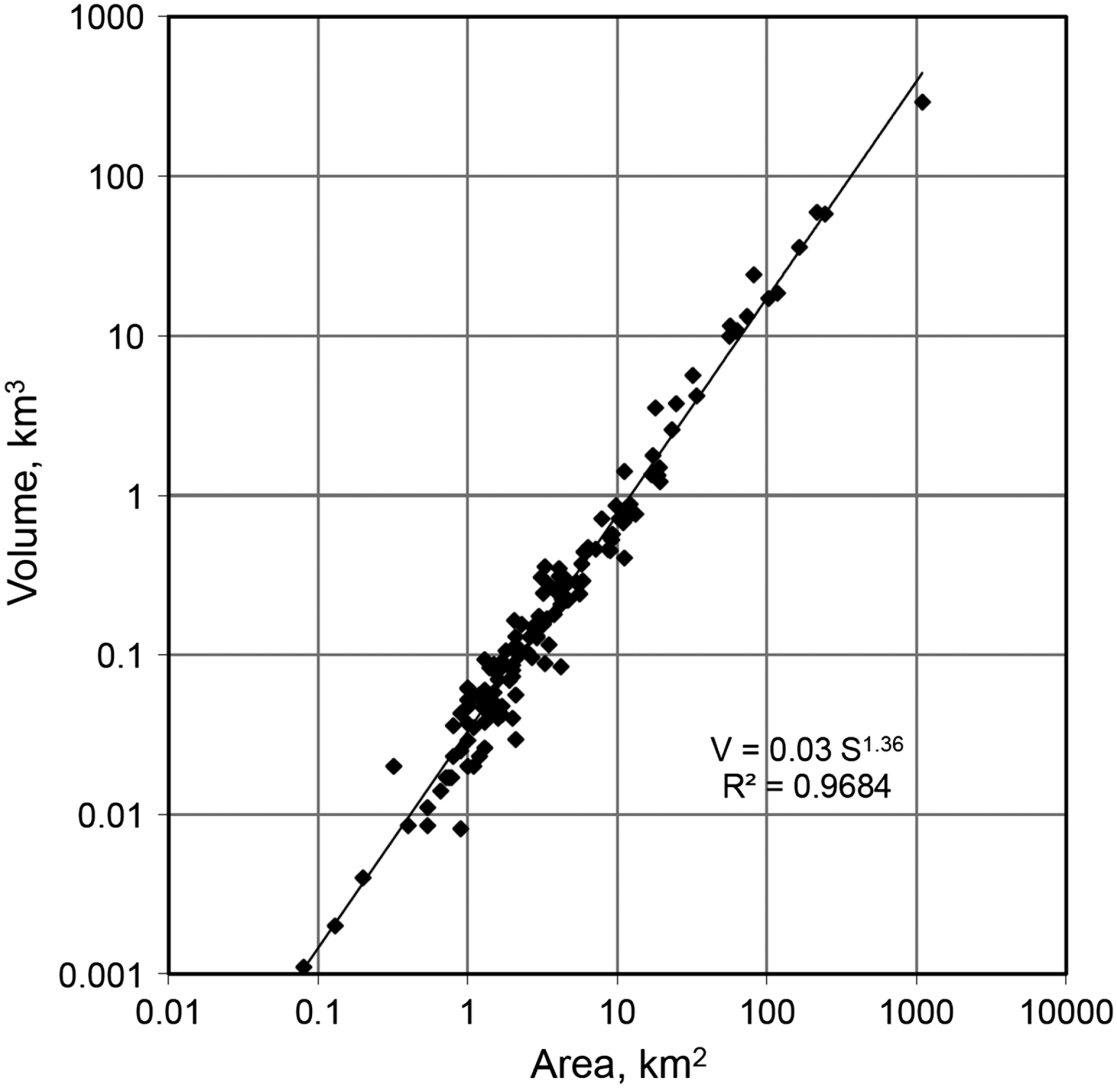
Glacier volume versus area for the 144 glaciers compiled in *Bahr et al*. [1997]. A power law relationship is clear with the linear trend on a log‐log plot. A simple linear regression suggests that the data are consistent with theory (*γ* = 1.375 in theory, and *γ* = 1.36 in this plot), a necessary though not sufficient condition for testing a power law hypothesis. The scaling hypothesis should also be tested with more sophisticated statistical techniques [*Clauset et al*., 2009] that are beyond the scope of this review but have been analyzed in detail elsewhere [*Grinsted*, 2013; *Bahr and Radić*, 2012]. Every effort was made to collect only those volumes that were measured rather than calculated. However, questions about the origins of volume data [*Haeberli et al*., 2007] emphasize the importance of having a rigorous physics‐based derivation of volume‐area scaling as presented in this paper. In the unlikely event that volume has been calculated from surface area for a majority of the measurements (see section 8.8), then a regression of V/S versus S might be used as a test of the scaling theory, but this alternative has other difficulties (Figure 2).

The key assumption in a traditional shallow‐ice approximation is that certain quantities identified by the dimensional analysis must be small [e.g., *Fowler and Larson*, 1978]. By relaxing this assumption, a more general set of scaling relationships can be derived. In the approach reviewed here, dimensionless quantities of any one glacier will be statistically similar but not necessarily identical to the dimensionless quantities of other glaciers. As long as the variance from glacier to glacier is small compared to the range of possible scaled values, then power law scaling relationships are discernable. For example, glacier areas around the world span 4 orders of magnitude and glacier volumes span over 5 orders of magnitude, but the variance of the relevant dimensionless quantities spans less than 1 order of magnitude, causing only the small amount of volume “noise” observed in Figure 1.

The practical distinction from previous shallow ice approximations is that the multiplicative parameter in a power law becomes a random variable [*Bahr*, 1997a] rather than a constant. The randomness comes from the statistically similar but not identical dimensionless parameters that vary from glacier to glacier. This difference has led to a number of misunderstandings and volume‐area scaling misapplications.

The literature review in Table 1 highlights many examples of both successful and unsuccessful scaling applications in glaciology. Generalized power law scaling is used most often to estimate sea level rise from changes in ice cap and glacier area, utilizing the so‐called volume‐area scaling relationship [e.g., *Raper and Braithwaite*, 2006; *Meier et al*., 2007; *Bahr et al*., 2009; *Leclercq et al*., 2011; *Radić and Hock*, 2011; *Slangen and van de Wal*, 2011; *Marzeion et al*., 2012; *Mernild et al*., 2013; among others]. A variety of scaling relationships have been used in other applications ranging from inversion theory [*Bahr et al*., 2014], to mass balance estimates [e.g., *Arendt et al*., 2006; *Barrand and Sharp*, 2010], to numerical modeling, where, for example, an unknown basal stress can be scaled with a more easily measured parameter such as glacier area [e.g., *Clarke et al*., 2013]. Response time scaling has been used to understand the basic response of glaciers to climate perturbations and has a long history in glaciology with notable papers by *Nye* [1960] and *Jóhannesson et al*. [1989], as well as more recent contributions by *Bahr et al*. [1998], *Pfeffer et al*. [1998], *Harrison et al*. [2001, 2003], *Lüthi* [2009], *Raper and Braithwaite* [2009], *Harrison* [2013], and others. As noted in many of these papers and in this review, the well‐established response time scaling and the newer volume‐area scaling relationship can be used and understood as closely related and mathematically intertwined concepts rooted in the same dimensional analysis. One does not exist without the other.

**Table 1:** A Summary of Selected Recent Studies Using Volume‐Area (V‐S) Scaling, Modifications of Volume‐Area Scaling, or Alternative Methods^a^

Reference		Application	Strengths/New Insights	Weaknesses/Errors^a^	Relevant Sections
*Adhikari and Marshall* [2012]		V‐S scaling evaluated in comparison to numerical model	Experiments compared to full 3‐D model; restriction to ensembles of glaciers (not individuals) recognized. Correctly identifies glacier shape and slope as drivers of volume (closure conditions in this work and in *Bahr et al*. [1997]).	Both *c* and *γ* treated as variables, or *c* is fixed and *γ* treated as variable; some derived values (e.g., *γ* > 2) fall outside theoretically meaningful limits; *Bahr et al*. [1997] V‐S scaling theory incorrectly claimed to be limited to shallow‐ice approximation and valid only for steady state conditions.	8.1, 8.2, 8.5, 8.9
*Agrawal and Tayal* [2013]		Volume change assessment in Himalayas.	Correctly specifies *γ* = 1.375 as a constant. Compares scaling results to other methods.	V‐S scaling applied to a single glacier.	8.2, 8.5
*Arendt*i [2006]		PhD thesis, volume changes of Alaskan glaciers	Scaling exponent *γ* treated as fixed at appropriate value; multiplier *c* as variable.	V‐S scaling hypothesized to be restricted to perfect plastic media. Time derivative incorrectly applied to V‐S scaling relationship with finite‐sized changes in area.	8.1, 8.7
*Arendt et al*. [2006]		Regional glacier mass balance assessment	Scaling exponent *γ* treated as fixed at appropriate value; multiplier *c* as variable. Uncertainty of application to single glaciers is recognized and discussed.	Time derivative incorrectly applied to V‐S scaling relationship. Analysis applied to single glacier.	8.1, 8.7,
*Barrand and Sharp* [2010]		Regional glacier mass balance assessment	Significant error in subdividing glaciers into subunits is recognized; very small fractions of glaciers considered are subdivided.	V‐S scaling exponent treated as variable; Scaling applied to artificial subunits of glaciers spanning political boundaries. Uncertainties judged relative to spurious error arising from assumed variability of scaling exponent.	8.2, 8.6
*Basagic and Fountain* [2011]		Volume estimates of small sample of glaciers	V‐S scaling recognized as highly uncertain for individual glacier volume estimate and characterized as “rough estimate.”	V‐S scaling used with a small sample (though correctly noted as a rough estimate).	8.5
*Binder et al*. [2009]		Ice volume estimate for two glaciers	Correctly specifies *γ* = 1.375 as a constant.	V‐S scaling applied to an individual glacier. Incorrectly specifies *c* = 1. Assumes V‐S theory is invalid for small retreating glaciers.	8.2, 8.5
*Bliss et al* [2013]		Regional glacier volume estimate	Proper values of *γ* for glacier/ice cap used except in case of BEDMAP (bed topography of the Antarctic) data only.	Assumes exponent *γ* is a variable with associated uncertainty; exponent *γ* treated as spatially variable. Values of *γ* for glacier/ice cap not distinguished when using BEDMAP data only.	8.2
*Farinotti et al*. [2009]		Global glacier volume estimate. Alternate method.	Method is applicable to single glaciers. An alternate numerical method for calculating volumes is compared to scaling.	V‐S scaling wrongly assumed to be limited to steady state conditions. V‐S scaling applied to individual glaciers.	8.1, 8.5
*Farinotti and Huss* [2013]		Estimates accuracy of V‐S scaling	Performs a rigorous statistical analysis of the accuracy of V‐S scaling. Correctly concludes that scaling should be applied to large populations of glaciers. Correctly notes that allowing *γ* to vary with time is less accurate than treating *γ* as a constant.	Assumes exponent *γ* is a spatial and temporal variable or a constant other than 1.375. Estimates *γ* from data. The variability of *γ* is built into the estimate of V‐S accuracy. A balance gradient is specified for the 3‐D Stokes model, but its value in equilibrium may be inconsistent with the V‐S closure condition.	8.2, 8.3, 8.7, 8.9, 8.11
*Fischer* [2009]		Ice volume estimate for single glacier	Recognizes that V‐S scaling should only be applied to populations of glaciers.	V‐S scaling applied to individual glaciers.	8.5
*Grinsted* [2013]		Global glacier volume estimate	Good compendium of previously published analyses; theoretical insights on role of surface slope; acknowledges that treating glacier complexes will degrade accuracy.	Attempts to assign multiple values to fixed exponent *γ* by empirical means and introduces additional parameters with no theoretical basis; treats glacier complexes as single entities.	8.2, 8.6, 9.1
*Haeberli et al*. [2007]		Regional/global glacier mass balance assessment	Points out that some glacier volume estimates may contain measure of area.	Claims invalidity of V‐S scaling on grounds that all volume measurements intrinsically contain measure of area.	7.1, 7.2, 8.8
*Hagg et al*. [2013]		Regional glacier mass balance assessment	Applicability of V‐S scaling to nonequilibrium conditions recognized.	V‐S scaling applied to sample of only 7 glaciers. Exponent *γ* treated as a variable with multiplier *c* held constant.	8.2, 8.4, 8.5
*Harrison* [2013]		Insights into the characteristic response of glaciers to climate	A macroscopic scaling‐type derivation with a nondimensional parameter consistent with this review. Correctly notes that the V‐S exponent is a constant. Combines volume scaling with response time scaling.	Theory is limited to an idealized geometry.	4.2, 8.4
*Huss and Farinotti* [2012]		Regional glacier volume estimate. Alternate method.	Method is applicable to single glaciers. V‐S scaling correctly noted to be inapplicable to multiple‐glacier complexes treated as single entity.	V‐S scaling wrongly assumed to be limited to steady state conditions and to be unsuitable for complex geometries such as dendritic glaciers. V‐S exponent treated as tunable parameter. V‐S exponent assumed spatially variable.	8.1, 8.2, 8.12
*Meehl et al*. [2007]		Global glacier mass balance assessment.	Scaling only applied to large populations of glaciers.	V‐S scaling wrongly assumed to be limited to steady state conditions	8.4
*Kulkarni and Karyakarte* [2014]		Regional glacier mass balance assessment	Calculates two different estimates of the volume for large populations of glaciers.	Polynomial volume‐area formula used without theoretical justification or discussion.	9.1
*Leclercq et al*. [2011]		Global historical glacier contribution to sea level.	Reconstruction of global glacier sea level contribution over past 2 centuries.	V‐S scaling wrongly assumed to be limited to single glaciers; method modified to extend to populations of glaciers.	8.5
*Lüthi* [2009]		Theoretical derivation of scaling parameters.	An alternative theoretical derivation of V‐S, volume‐length, and response time scaling for a limited geometry. Results are largely consistent with this review and with a 3‐D Stokes model. Contains a theoretical derivation of *c* for the specified geometry. Correctly applies response time and V‐S scaling simultaneously.	Theory is limited to an idealized geometry.	7.1, 7.2, 8.4, 8.10
*Marzeione et al*. [2012]		Scaling applied in numerical model of future global glacier mass balance.	Robust treatment of time scale of transients. Proper scaling parameters adopted.		8.2, 8.4
*Moore et al*. [2013]		Overview of projection methods for glacier mass balance	Recognizes that V‐S scaling cannot be applied to multiple‐glacier complexes treated as single entity or to subunits of a glacier.	V‐S scaling parameters wrongly assumed to depend on assumption of perfect plasticity and to be intrinsically variable.	8.1, 8.6
*Möller and Schneider* [2010]		Glacier mass balance modeling	Parameter *γ* correctly assumed to be a constant. Single‐glacier limitation avoided by using data for the same glacier at multiple times.	Parameter *c* = 1 incorrectly assumed to hold for equilibrium conditions; empirical value for *c* ≠ 1 sought for nonequilibrium conditions.	7.1, 7.2, 8.2
*Radić and Hock* [2010]		Global glacier volume estimate	Selects appropriate values for *γ* for glaciers and for ice caps.	Assumes parameter *γ* has associated uncertainty.	8.2
*Radić and Hock* [2011]		Global glacier volume estimate	Selects appropriate values for *γ* for glaciers and for ice caps; notes potential time variation in parameter *c*.	Assumes parameter *γ* has associated uncertainty.	8.2
*Radić et al*. [2007]		V‐S scaling exponents derived by numerical model	Acknowledges potential for errors from use of 1‐D model.	A 1‐D glacier model is used (V‐S parameters derived from this model will not necessarily apply to real glaciers); unrealistically wide range of values for parameter *γ* found. Assumes *γ* is time dependent for nonequilibrium conditions. Assumes V‐S scaling applies only to steady state conditions.	8.1, 8.2, 8.3, 8.9
*Radić et al*. [2008]		V‐S scaling evaluated in comparison to numerical model	V‐S scaling adapted to nonsteady conditions; application of multiple scaling relationships to single glacier.	Parameter *γ* treated as adjustable parameter; assumes parameter *γ* must vary to account for nonequilibrium conditions.	8.2, 8.4
*Raper and Braithwaite* [2006]		Global glacier volume estimate	One of the first papers to pioneer the use of V‐S scaling to estimate global glacier ice volume.	Treats glacier complexes as single entities; subdivides glacier along grid cell boundaries and applies scaling to subunits.	8.6
*Salzman et al*. [2013]		Regional glacier volume estimate	V‐S scaling properly applied to aggregate glacier in Peruvian Andes. Uses scaling as a test of a numerical model.		8.10
*Schneeberger et al*. [2003]		GCM‐forced regional/global glacier mass balance	Recognizes that V‐S scaling should only be applied to populations of glaciers.	V‐S scaling applied to individual glaciers.	8.5
*Slangen and van de Wal* [2011]		Uncertainty assessment of V‐S scaling in sea level rise projections	Fixes exponent *γ* at appropriate values for glaciers (*γ* = 1.375) and ice caps (*γ* = 1.25). Allows *c* to vary.	Incorrectly performs a sensitivity analysis on *γ* which should remain constant.	8.2
*van de Wal and Wild* [2001]		Glacier mass balance model	One of the first papers to pioneer the use of V‐S scaling to estimate sea level. Fixes exponent *γ* at appropriate values for glaciers (*γ* = 1.375) and ice caps (*γ* = 1.25); recognizes that V‐S scaling applies in nonequilibrium conditions	Incorrectly performs a sensitivity analysis on *γ* which should remain constant.	8.2
*Wang et al*. [2014]		Ice volume estimate for single glacier	Recognizes that V‐S scaling is highly uncertain when applied to single glacier.	V‐S scaling applied to single glacier. Scaling exponent assumed to depend on individual glacier characteristics and time.	8.2

a In some cases, the identified error or weakness only propagates a common misunderstanding without detracting from the overall value of the study. In many cases, the insights of the paper remain substantial even though the conclusions may need revisiting. In a few cases, the errors would warrant a careful reevaluation of the conclusions.

Because it is the most commonly used scaling relationship, we present a comprehensive derivation of volume‐area scaling, starting with a complete dimensional analysis of the full set of stress, continuity, and constitutive equations in three dimensions without simplifications. The resulting dimensional relationships have value to glaciology beyond simply relating volume to area; for example, the dimensionless parameters for glaciers can be used to rescale a numerical model using the principles of dynamic similitude [e.g., *Baral et al*., 2001; *Bahr and Rundle*, 1995] to evaluate response times [*Jóhannesson et al*., 1989], or to construct new scaling relationships [*Bahr*, 1997a].

As in heat and mass transfer, dimensional relationships for glaciers can in some instances be derived informally by inspection of the equations, simply taking those ratios of terms that yield dimensionless values. However, to avoid ambiguity, we present a rigorous derivation based on both stretching symmetries and the Buckingham Pi method. The Buckingham Pi Theorem is both simpler and sufficient but assigns no physical meaning to the resulting dimensionless parameters (for example, the Buckingham method does not make it immediately obvious if a dimensionless parameter comes from conservation of mass, momentum, energy, or none of the above). Stretching symmetries, on the other hand, are based on the requirement that rescaled independent variables must yield compatible rescaled dependent variables for any particular governing equation. Stretching thus ties each dimensionless parameter to a specific equation. The details are rooted in Lie group theory [*Logan*, 2004], but the application of stretching is straightforward and can be understood in the context of this review without reference to the underlying theory. Both stretching and the Buckingham Pi Theorem give identical results.

## 2 Fundamental Dimensional Principles

Volume‐area scaling provides a very simple and robust method for estimating glacier volume from observations of area. For glacier area *S* the volume *V* can be estimated as

(1) $$V=cS^{\gamma }$$

where the parameters *c* and *γ* were originally determined as empirical constants [*Macheret et al*., 1988; *Chen and Ohmura*, 1990; *Zhuravlev*, 1988]. Additional data were later compiled by *Meier and Bahr* [1996], *Bahr et al*. [1997], *Cogley* [2010], and *Grinsted* [2013] (Figure 1). Equation (1) provides a simple estimate of glacier volume, a quantity that is very difficult to measure, in terms of glacier area, which can be measured very easily. However, the power law relationship of equation (1) was originally established on a purely empirical basis, and some theoretical justification is needed if this relationship is to be assumed valid under circumstances different from those used to establish the relationship and quantify the parameters initially (e.g., such as future environmental conditions, significantly larger and smaller glaciers, and equilibrium versus nonequilibrium conditions). This was established by *Bahr et al*. [1997] and *Bahr* [1997a].

**Figure 2:**
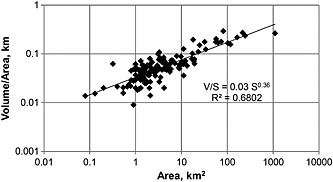
Average glacier thickness approximated as the volume divided by area. For the 144 glaciers compiled in *Bahr et al*. [1997], the data show an apparent power law trend between the thickness and the area. A simple regression is consistent with the theory (*γ* = 1.375 in theory, and *γ* − 1 = 0.36 in this plot), a necessary though not sufficient condition for testing a power law hypothesis. The scaling hypothesis should also be tested with more sophisticated statistical techniques [*Clauset et al*., 2009] that are beyond the scope of this review but have been analyzed in detail elsewhere [*Grinsted*, 2013; *Bahr and Radić*, 2012]. However, with less than 2 orders of magnitude in thickness in this plot, noise becomes problematic and a power law hypothesis is difficult to assess (see the theoretical reasons for this noise at the beginning of section 7). The general scaling theory might be better tested with one of the many alternative scaling relationships derived in the text (e.g., equation (138)) and elsewhere [e.g., *Bahr*, 1997a].

Despite the simplicity of equation (1), considerable care and insight are required to apply it correctly. Although simple in form, equation (1) contains many subtleties that can only be understood in terms of the underlying theory and that have been overlooked or incorrectly interpreted in various instances in the published literature. For example, while theory predicts that the scale factor *c* is a random variable and the exponent *γ* is a constant for a given geometric class of glacier (specifically valley glaciers as opposed to radially symmetric ice caps—see sections 7.1 and 7.2), applications have often and incorrectly derived regionally and temporally varying exponents and incorrectly assumed constant scale factors [e.g., *Adhikari and Marshall*, 2012; *Grinsted*, 2013]. Similarly, the theoretical basis of equation (1) is determined for samples of glaciers spanning a wide range of sizes (and is validated by observations covering a wide range of sizes) but has been frequently assumed to apply to individual glaciers [e.g., *Binder et al*., 2009]. Volume‐area scaling can in fact be applied to individual glaciers, but the individual glacier volume so determined will be only order‐of‐magnitude accurate, a fact that is clear from an inspection of any plot of empirically derived values (Figures 1 and 2). However, in one case [*Leclercq et al*., 2011], the theory was mistakenly adjusted further to accommodate the application to more than one glacier, having incorrectly assumed the original theory applies *only* to individual glaciers. Furthermore, volume‐area scaling cannot be used on parts or individual branches of a glacier nor will it work well for glacier complexes that span a flow divide, draining to multiple outlets; and while numerical modeling approaches have many advantages (e.g., the ability to calculate additional parameters such as velocities), numerical models cannot improve estimates of the *aggregate* volume of collections of glaciers over that derived by volume‐area scaling [*Bahr et al*., 2014], despite occasional claims to the contrary [e.g., *Haeberli et al*., 2007].

The validity of these and other limitations (and strengths) are rooted in the underlying mechanics which are, for better or worse, hidden by equation (1), but which are explained in *Bahr et al*. [1997] and extended in *Bahr* [1997a]. Without the theoretical underpinning, volume‐area data could only be tested as a statistical hypothesis, and there would always be a concern that another functional relationship could be a better fit to the data, or that conditions might exist for which equation (1) would be invalid. With the theoretical foundation, volume‐area data become a test of the theory itself, and the power law relationship can be applied with confidence. There is no longer a question, for example, that the volume‐area relationship might fail at small or large glacier sizes that fall outside of the observed data. Theory says it would not.

Scaling relationships in glaciology and in many other natural sciences can often be derived from a simple dimensional analysis of the physical quantities. Power law relationships between these quantities are the natural and only form of a dimensionless parameter [*Buckingham*, 1914; *Schmidt and Housen*, 1995], and as a consequence, power laws occur as relationships between significant properties characterizing natural systems with striking regularity [*Dodds and Rothman*, 2000]. Consider, for example, the Reynolds number *Re* of fluid dynamics, which describes the relative importance of momentum and viscosity in fluid flow [*Welty et al*., 1984]:

(2) $$Re=\frac{vl}{\upsilon }$$

where *v* is fluid velocity, *l* is length, and *υ* is kinematic viscosity. Rearranging gives a scaling relationship between velocity and length:

(3) $$v=\left(\upsilon Re\right)l^{-1}$$

where the scaling exponent is −1 and the multiplicative scaling parameter is *υRe*. Note that the scaling parameter *υRe* is not a constant because *Re* has a distribution of possible values. In contrast, the scaling exponent −1 *is* a constant, fixed by the dimensional analysis. The exponent is determined by the algebraic structure of the dimensionless parameter, and not by magnitudes characterizing the process.

The scaling exponent is not always an integer. For example, the Froude number *Fr* of fluid mechanics relates fluid velocity to gravity *g* and the square root of length [*Welty et al*., 1984]. Rearranging terms of that expression gives

(4) $$v=\left(Fr\;g^{\frac{1}{2}}\right)l^{\frac{1}{2}}$$

Again, the power law scaling exponent of 1/2 is fixed by the physics, but the multiplicative scaling parameter *Fr g* ^1/2^ is variable because the Froude number can have a distribution of possible values.

Combinations of dimensionless parameters can lead to new power law scaling relationships. From the dimensionless Euler number *Eu*, velocity is inversely related to the square root of pressure *P* and density *ρ*:

(5) $$v={\left(\rho Eu\right)}^{-1/2}P^{1/2}$$

Combining with (3) gives a new power law scaling relationship between pressure and length.

(6) $$P=\left(\rho \upsilon^{2}Re^{2}Eu\right)l^{-2}$$

In this case the immutable scaling exponent is fixed at −2, and the mutable multiplicative scaling parameter depends on two variable quantities, *Re* and *Eu*.

Glacier volume‐area scaling adheres to the same dimensional principles as these examples from fluid mechanics. A dimensional analysis is used to derive general scaling relationships applicable to glaciology, and as always, the exponents are constants while the multiplicative scaling parameters are not. The volume‐area scaling relationship is then formed from a combination of these general dimensionless parameters. Although there are important subtleties explored below (see section 8), the volume‐area scaling exponent *γ* of equation (1) is a constant and the volume‐area scaling parameter *c* is a variable that depends on combinations of several variable dimensionless parameters that are analogous, in the case of creeping ice flow, to the Reynolds, Froude, and Euler numbers for the flow of fluids like water. It makes no sense to talk about alternative exponents or regionally applicable exponents or time‐dependent exponents for the volume‐area scaling relationship. Just as the exponents in Reynolds number are fixed by the physics, so is *γ* fixed by the physics of flowing ice.

Furthermore, in order for two Newtonian fluid flows to be “similar” in the mathematical sense (for example, in a modeled flow and in a full‐scale flow), they must have the same geometry and all of the same Reynolds, Froude, and Euler numbers; if any one of these numbers are different, then the flows will be fundamentally different. The same applies to ice flow, and this will be especially relevant for time‐dependent behaviors. Volume‐area scaling and response time scaling are both well‐known glaciological relationships, but one cannot be considered separately from the other. Instead, a real glacier is characterized and modeled by both relationships simultaneously, and there is no need to artificially assign time dependence to the volume‐area exponent.

A number of publications have explored potential modifications and variations of the glacier volume‐area scaling relationship, including time dependence. As discussed below in section 9, some of these modifications may have a sound basis in the underlying scaling physics. Modifications that consider vertical glacier range and slope are two examples, if they are handled in a very specific manner that adheres to dimensionless principles. Many studies have made valuable contributions that are largely consistent with scaling theory, using volume‐area to estimate aggregate ice volumes [*Radić and Hock*, 2010; *Grinsted*, 2013] to predict future changes in ice mass [*Meier et al*., 2007; *Leclercq et al*., 2011; *Bahr et al*., 2009; *Mernild et al*., 2013; *Radić and Hock*, 2011; *Radić et al*., 2013; *Raper and Braithwaite*, 2006; *Marzeion et al*., 2012] to explore the sensitivity of volume‐area scaling to perturbations [*Van de Wal and Wild*, 2001; *Slangen and Van de Wal*, 2011] and to understand related scaling relationships, particularly the volume response time [*Jóhannesson et al*., 1989; *Bahr et al*., 1998; *Pfeffer et al*., 1998; *Raper and Braithwaite*, 2009]. On the other hand, many of the proposed modifications and a number of related applications have been in direct conflict with the underlying theory. A partial list of examples includes improper time dependence [*Arendt et al*., 2006; *Huss and Farinotti*, 2012; *Möller and Schneider*, 2010], inappropriate scaling parameters [*Grinsted*, 2013; *Hagg et al*., 2013; *Möller and Schneider*, 2010], assumptions of steady state and/or shallow ice [*Huss and Farinotti*, 2012; *Möller and Schneider*, 2010; *Meehl et al*., 2007], applications to individual glaciers without regard to accuracy [*Agrawal and Tayal*, 2013; *Schneeberger et al*., 2003], applications to portions of glaciers or to undifferentiated glacier complexes [*Grinsted*, 2013; *Raper and Braithwaite*, 2006], and a general rejection of volume‐area scaling based on inappropriate statistical arguments [*Haeberli et al*., 2007]. These conflicts will be discussed further below, and Table 1 highlights a partial list of published volume‐area applications along with their most prominent contributions and occasional inconsistencies with established theory.

### 2.1 Basic Derivation

Volume can be scaled with many quantities, including, but not limited to, glacier area, thickness, length, response time, and velocity [*Bahr*, 1997a]. Of the many possibilities, the surface area is perhaps easiest to measure making it a practical parameter for estimating glacier volume or other properties. Some published analyses choose instead to scale area with thickness rather than area with volume [*Cogley*, 2012]. This is a theoretically sound approach that reflects an important and fundamental relationship. However, many if not most published studies need estimates of glacier volume rather than estimates of thickness, and as shown below, multiplying the thickness by area may lead to an estimate of the volume that does not properly account for valley cross sections.

The volume‐area scaling relationship is derived in full detail in the later sections, but the basic principle for small glaciers is described in compact form here and can be generalized to other relationships. Because volume *V* has dimensions of length cubed (*L* ^3^), and surface area *S* has dimensions of length squared (*L* ^2^), a dimensional analysis can relate *V* to *S* by multiplying the area *S* by one additional quantity with dimensions of length (*L*).

Glacier thickness is an obvious choice for the additional dimension of *L* because surface area already contains measures of width and length. However, we must adjust the centerline thickness *h* with a shape factor *F* to account for drag along valley side walls [e.g., *Nye*, 1965; *Cuffey and Paterson*, 2010, p. 342]:

(7) V∝S⋅Fh

The area *S* is a product of length *l* and width *w*. The shape factor *F* scales as a ratio of width to thickness [*Bahr et al*., 1997, equation (12)]. (This scaling works for smaller values of *F* and is appropriate for many valley glaciers [*Bahr et al*., 1997], but ice caps and larger values of *F* will require a different derivation, as detailed later.) Therefore,

(8) $$\begin{array}{c} V\propto wl\cdot \frac{w}{h}h\\ =w^{2}l \end{array}$$

Data show that width scales with length as

(9) $$w\propto l^{q}$$

where *q* ≈ 0.6 = 3/5 [*Bahr*, 1997b]. Therefore,

(10) $$S\propto wl=l^{q+1}$$

Substituting (10) into (8) gives

(11) $$\begin{array}{c} V\propto w^{2}l\\ \propto l^{2q+1}\\ \propto S^{\frac{2q+1}{q+1}}\\ \propto S^{1.375} \end{array}$$

Some specific boundary or “geometric closure” condition is necessary to fully quantify the relationship being expressed. The closure conditions are imposed scaling relationships (rather than imposed continuum mechanical boundary conditions) that apply on average to all glaciers. In this case the closure condition is *q =* 0.6, entering through the width‐length scaling equation (9). In the following more detailed derivations, we demonstrate that we could instead use data from mass balance scaling or from equilibrium accumulation area ratios (the ratio of the accumulation area to the total area for a glacier in dynamic equilibrium). All three closures give the same result, and, reassuringly, each closure can be derived from the other.

While the above derivation is straightforward, it relies on physical intuition for the choice of *Fh* as a length scale, and a different choice of this important but unobvious factor could yield a different result. This requirement of physical insight is typical of dimensional analyses, but the following more detailed “directional” dimensional analysis, shown below, makes the choice of *h* inevitable. In essence, all three coordinate directions must be represented, and *S* already contains the other two coordinate directions within the length *l* and width *w*.

### 2.2 An Outline of the Complete Derivation

A fully detailed volume‐area scaling derivation requires a dimensional analysis of all the pertinent glaciological variables. In the following, section 3 highlights the relevant continuum equations and therefore the relevant variables as well. Section 4 then uses a dimensional analysis (the Buckingham Pi Theorem) to derive a set of dimensionless parameters that are used in a later section to construct the volume‐area relationship. A directional analysis is then used to refine the dimensionless parameters; to our knowledge, a directional analysis has not been previously used in glaciology. The advantage of the combined dimensional and directional analyses is that the final results are independent of the underlying flow equations, whether the flow equations originate from continuum mechanics, more generally from statistical mechanics (e.g., Boltzmann simulations of glacier flow [*Bahr and Rundle*, 1995]), or from alternatives such as discrete representations with cellular automata [e.g., *Frisch et al*., 1986].

The results of the dimensional analysis in section 4 can also be obtained independently by using a stretching symmetry transformation of the continuum equations. Section 5 details the stretching symmetry analysis, and the results are seen to be identical to the dimensional analysis in section 4. This is both reassuring (two completely independent techniques give the same answer) and helpful because the source of each dimensionless parameter and scaling relationship can now be traced to its origin in the continuum equations.

The long derivations of section 5 can be skipped if desired, but numerical modelers may find it particularly helpful because it shows how to take an arbitrary set of differential equations and scale them appropriately. For example, if a glacier flow model uses a modified or simplified set of continuum equations (e.g., restrictions to plane strain, shallow ice, or vertically integrated continuity without the full three‐dimensional force balance), then the scaling relationships that apply to these modified equations would need to be rederived using the stretching techniques described in section 5. There is no guarantee that a simplified or otherwise modified set of glacier continuum equations will scale in the same manner as the complete set of equations. As such, section 5 is essential for numerical modelers that want to compare their results to volume‐area scaling.

Section 6 summarizes the full set of dimensionless parameters derived and outlines how to choose and use characteristic values. The process is similar to choosing characteristic values in the more familiar context of fluid mechanics with the Reynolds, Froude, and Euler numbers. The correct choice depends on the application.

Section 7 then completes the volume‐area scaling analysis by using appropriate characteristic values to give a complete derivation of both the volume‐area scaling exponent *γ* and the scaling parameter *c*. A derivation of the scaling parameter *c* has not been previously published. Section 7 also reduces the number of necessary closure conditions to just one (three closures were used in *Bahr et al*. [1997]).

While the exposition may be long, our hope is that this unabbreviated derivation can be used as a baseline for future studies while eliminating many of the misconceptions about scaling in general and volume‐area scaling in particular that appear in the glaciological literature. Those desiring a shorter and abbreviated discussion of the volume‐area scaling derivation are referred to *Bahr* [1997a, 2011]. A shorter and simpler two‐dimensional discussion of the dimensional analysis can be found in *Bahr and Rundle* [1995].

## 3 Continuum Equations

The creeping flow of ice is completely described by continuum mechanics with the continuity equation (mass balance and conservation of mass), the equations of motion (force balance and conservation of momentum), and the constitutive equation (linking mass and momentum conservation by relating force to deformation or stress to strain rate). Conservation of energy (temperature of ice) can be included as well (see section 4.1) but has no direct effect on the volume‐area scaling equation (1). Instead, the inclusion of energy balance introduces three additional dimensionless parameters without altering the other parameters.

From conservation of mass, the continuity equation is

(12) $$-\frac{\partial \rho }{\partial t}=\frac{\partial }{\partial x}\left(\rho \;u_{x}\right)+\frac{\partial }{\partial y}\left(\rho \;u_{y}\right)+\frac{\partial }{\partial z}\left(\rho \;u_{z}\right)+\dot{\mu }$$

where $\dot{\mu }$ is the rate of mass gained per unit volume (rate of change in density due to a source of new ice). Ice is incompressible, so this equation is often integrated over the glacier thickness (with *z* typically selected as the vertical axis) to give

(13) $$\frac{\partial h}{\partial t}=\dot{b}-\frac{\partial }{\partial x}\int_{h_{b}}^{h_{s}}u_{x}dz-\frac{\partial }{\partial y}\int_{h_{b}}^{h_{s}}u_{y}dz$$

[e.g., *Cuffey and Paterson*, 2010], where the ice thickness *h* is related to the surface elevation *h_s_* and bed elevation *h_b_* as

(14) $$h=h_{s}-h_{b}$$

Englacial sources or sinks of ice are included as $\dot{\mu }$ in (12), but in (13) they are included as part of $\dot{b}$, which can be defined as the total mass accumulation rate over the thickness of the glacier. Generally, internal melting and accumulation of ice is negligible compared to surface processes, and $\dot{b}$ is typically assumed to be only the surface mass balance rate. We do not make that assumption in this analysis, and we do a stretching analysis of both (12) and (13) to show that the results are the same.

The equations of motion include conservation of momentum:

(15) $$\frac{\partial \sigma_{ii}}{\partial x_{i}}+\frac{\partial \sigma_{ij}}{\partial x_{j}}+\frac{\partial \sigma_{ik}}{\partial x_{k}}+\rho g_{i}=\rho \frac{Du_{i}}{Dt}$$

where *i*, *j*, *k* ∈ {*x*, *y*, *z*}, *i* ≠ *j* ≠ *k*, and we use *x_i_* to indicate the coordinate directions *x*, *y*, or *z*. For clarity in later derivations, we do not use the Einstein summation notation. Note that D/D*t* is the material derivative:

(16) $$\frac{D}{Dt}=\frac{\partial }{\partial t}+u_{x}\frac{\partial }{\partial x}+u_{y}\frac{\partial }{\partial y}+u_{z}\frac{\partial }{\partial z}$$

For glaciers, acceleration is negligible, and the right‐hand side of (15) is typically set to zero. In that case, equation (15) becomes the static stress equilibrium equations.

Stresses and strain rates are related by a constitutive relation. Like the vast majority of glaciological publications, we assume a standard Glen's flow law [e.g., *Cuffey and Paterson*, 2010]. This widely used and widely accepted relationship is the only assumption in our dimensional analysis. The dimensional parameters could change if another constitutive relationship were hypothesized. For *i*, *j* ∈ {*x*, *y*, *z*},

(17) $${\dot{\varepsilon }}_{ij}=A{\left(\frac{1}{2}\sum_{i,j}\sigma_{ij}^{\prime }\sigma_{ij}^{\prime }\right)}^{\frac{n-1}{2}}\sigma_{ij}^{\prime }$$

where the stress deviators are defined as

(18) $$\begin{array}{c} \sigma_{ij}^{\prime }=\sigma_{ij}\\ \sigma_{ii}^{\prime }=\sigma_{ii}-\frac{1}{3}\left(\sigma_{xx}+\sigma_{yy}+\sigma_{zz}\right) \end{array}$$

and strain rates are defined as

(19) $$\begin{array}{c} {\dot{\varepsilon }}_{ij}=\frac{1}{2}\left(\frac{\partial u_{i}}{\partial x_{j}}+\frac{\partial u_{j}}{\partial x_{i}}\right)\\ {\dot{\varepsilon }}_{ii}=\frac{\partial u_{i}}{\partial x_{i}} \end{array}$$

Boundary conditions specify the geometry, the mass balance along each surface, a zero traction condition at the surface, and a specified stress or sliding velocity at the bed. These boundary conditions have no direct effect on the dimensional analysis that follows. A dimensional analysis depends on the dimensions of the underlying variables rather than the manner in which these variables appear in a specific equation. Boundary conditions do not change the dimensions of the variables. If new continuum variables are introduced through boundary conditions, then this will create additional dimensionless parameters, but it does not alter the other dimensionless parameters previously derived without these new variables. Basal sliding laws in particular can introduce new parameters and variables associated with till constitutive properties, bed roughness, etc. These new parameters might even depend on glacier size (volume and surface area) by increasing the temperature at the bed and consequently increasing till creep and slip. However, this will not directly affect the volume‐area scaling relationship because the volume‐area power law is derived from the dimensionless parameters associated with the variables already present in equations (12), (13), (14), (15), (16), (17), (18), (19).

## 4 Dimensional Analysis and Buckingham Pi

### 4.1 Dimensional Analysis

An inspection of the previous equations shows that there are 18 fundamental variables, *ρ*, *t*, *x*, *y*, *z*, *u_x_*, *u_y_*, *u_z_*, *g_x_*, *g_y_*, *g_z_*, *σ_xx_*, *σ_xy_*, *σ_xz_*, *σ_yy_*, *σ_yz_*, *σ_zz_*, and *A*. An argument could be made that thickness *h* should be included, but both *z* and *h* measure a quantity along the same vertical axis. Therefore, we choose only the more general variable *z* to avoid repetition. Similarly, we have chosen the more general vertical velocity *u_z_* rather than surface mass balance rate $\dot{b}$. As discussed above, introducing specific boundary conditions like basal sliding can introduce new variables to this list, but as will be seen with the inclusion of the energy balance equation at the end of this section, new variables introduce new dimensionless parameters without altering the existing parameters.

We know that any physically plausible relationships between these 18 variables must be dimensionally consistent. This forms the basis of the Buckingham Pi Theorem which derives all possible dimensional parameters from the relevant variables without any knowledge of the underlying equation(s). The Buckingham Pi Theorem states that if there are *N* variables (e.g., velocity) composed of *P* fundamental dimensions (e.g., mass, length, time, and temperature), then there are a total of *N – P* possible dimensionless parameters [*Schmidt and Housen*, 1995; *Welty et al*., 1984; *Buckingham*, 1914]. In this case there are 18 variables with three fundamental dimensions (mass *M*, length *L*, and time *T*), so there are 18 – 3 = 15 dimensionless parameters that describe glacier flow.

Table 2 shows a dimensional matrix for the 18 variables where each row is a fundamental dimension, and each column indicates the number of dimensional units contained by a particular variable. For example, density *ρ* has dimensions of *M*/*L* ^3^ where *M* represents units of mass and *L* represents units of length. Following a general Buckingham method, we choose three variables (columns in the table) whose units span all of the *P* dimensions. These particular variables can be variously combined to construct the dimensions of any other variable.

**Table 2:** A Dimensional Matrix Showing the Relationship Between Each Continuum Variable and Its Fundamental Dimensions (*L* for Length, *T* for Time, and *M* for Mass)^a^

	*ρ*	*t*	*x*	*y*	*z*	*u_x_*	*u_y_*	*u_z_*	*g_x_*	*g_y_*	*g_z_*	*A*	σ*_xx_*	σ*_xy_*	σ*_xz_*	σ*_yy_*	σ*_yz_*	σ*_zz_*
*L*	–3	0	1	1	1	1	1	1	1	1	1	*n*	–1	–1	–1	–1	–1	–1
*T*	0	1	0	0	0	–1	–1	–1	–2	–2	–2	2*n* – 1	–2	–2	–2	–2	–2	–2
*M*	1	0	0	0	0	0	0	0	0	0	0	–*n*	1	1	1	1	1	1

a From the Buckingham Pi Theorem, the rank of the dimensional matrix gives the minimum number of variables necessary to reproduce the dimensional structure of all other variables. For this matrix, the rank is three. By inspection, the three columns associated with *ρ*, *t*, and *x* are independent and contain all three dimensions. These three columns form a set of basis vectors that can be used to construct the dimensions of each of the other variables. Other basis vectors are obvious from the table (e.g., *A*, *ρ*, and *z*), but a different choice gives only a recombination of the dimensionless parameters predicted by the original choice. For details about dimensional matrices, we recommend *Welty et al*. [1984].

An inspection of Table 2 shows that we can select density *ρ*, time *t*, and length *x* as representative variables that span all of the dimensional units (*M*, *L*, *T*). Now consider the variable *y*. A dimensionless parameter Π*_y_* can be constructed from *ρ*, *t*, *x*, and *y* as

(20) $$\Pi_{y}=\rho^{a}t^{b}x^{c}y$$

for some *a*, *b*, and *c*. Clearly, *a* = 0, *b* = 0, and *c* = −1 are the only dimensionally consistent choices. In other words,

(21) $$\Pi_{y}=\frac{y}{x}$$

This is the same process as the familiar nondimensionalization in which all length scales are represented by their ratio with some selected reference length scale. Similarly, for the horizontal velocity *u_x_*,

(22) $$\Pi_{u_{x}}=\rho^{a}t^{b}x^{c}u_{x}$$

The only possible exponents are *a* = 0, *b* = 1, and *c* = −1, so

(23) $$\Pi_{u_{x}}=\frac{t\;u_{x}}{x}$$

The process can be repeated for every variable to give all 15 dimensionless parameters. For more details of the methodology, see *Welty et al*. [1984].

Note that new and different dimensionless parameters can be constructed by substitution. For example, a new parameter $\Pi_{u_{x}y}$ can be constructed by dividing (23) with (21).

(24) $$\Pi_{u_{x}y}=\frac{\Pi_{u_{x}}}{\Pi_{y}}=\frac{t\;u_{x}}{y}$$

Clearly, this parameter is not independent of the others. No matter how they are constructed, the Buckingham Pi Theorem indicates that there can only be 15 independent dimensionless parameters.

After some simplification and substitutions, the following lists all 15 independent dimensionless numbers associated with glacier flow.

(25) $$\Pi_{1}=\frac{x}{y}$$

(26) $$\Pi_{2}=\frac{x}{z}$$

(27) $$\Pi_{3}=\frac{u_{x}}{u_{y}}$$

(28) $$\Pi_{4}=\frac{u_{x}}{u_{z}}$$

(29) $$\Pi_{5}=\frac{t\;u_{x}}{x}$$

(30) $$\Pi_{6}=\frac{g_{x}}{g_{z}}$$

(31) $$\Pi_{7}=\frac{g_{y}}{g_{z}}$$

(32) $$\Pi_{8}=\frac{t^{2}g_{z}}{z}$$

(33) $$\Pi_{9}=\frac{A\rho^{n}{g_{x}}^{n}z^{n+1}}{u_{x}}$$

(34) $$\Pi_{10}=\frac{\rho g_{x}z}{\sigma_{xz}}$$

(35) $$\Pi_{11}=\frac{\sigma_{xz}}{\sigma_{xx}}$$

(36) $$\Pi_{12}=\frac{\sigma_{xz}}{\sigma_{xy}}$$

(37) $$\Pi_{13}=\frac{\sigma_{xz}}{\sigma_{yy}}$$

(38) $$\Pi_{14}=\frac{\sigma_{xz}}{\sigma_{yz}}$$

(39) $$\Pi_{15}=\frac{\sigma_{xz}}{\sigma_{zz}}$$

We might reasonably argue that stresses are redundant and implied by changes in velocity via strain rates (the constitutive equation). In that case there are only 12 fundamental variables, *t*, *x*, *y*, *z*, *u_x_*, *u_y_*, *u_z_*, *g_x_*, *g_y_*, *g_z_*, *ρ*, and *A*. By the same process as before, Buckingham Pi implies there are 12 – 3 = 9 independent dimensionless parameters which are given by Π_1_ through Π_9_ in equations (25), (26), (27), (28), (29), (30), (31), (32), (33).

Although it does not change our derivations, for completeness we note that including conservation of energy would introduce four fundamental variables: temperature Φ (units of K), thermal conductivity *k* (units kg m/(s^3^ K)), volumetric rate of thermal energy production *f* (units kg m/s^3^), and the (usually negligible) viscous work rate per unit volume *w_v_* (units kg m^2^/s^2^) [*Welty et al*., 1984, p. 245]. That gives 22 fundamental variables, 4 fundamental units (with the addition of temperature), and 22 – 4 = 18 dimensionless parameters. Including Φ, *f*, *k*, and *w_v_* to the dimensional matrix in Table 2, it is obvious that none of Π_1_ to Π_15_ will be changed. Instead, three new dimensionless parameters are generated:

(40) $$\Pi_{16}=\frac{t^{3}\Phi k}{\rho x^{4}}$$

(41) $$\Pi_{17}=\frac{t^{2}f}{\rho x^{4}}$$

(42) $$\Pi_{18}=\frac{t^{2}w_{v}}{\rho x^{5}}$$

Note that although the parameter *A* depends on temperature via the exponential Arrhenius relationship, the units of *A* do not depend on temperature. Therefore, the inclusion of temperature does not change any of the dimensional relationships that include *A*. Although we will not use Π_16_, Π_17_, or Π_18_ again in this review, note that the relationship between temperature, time, and length in (40) could be used to derive interesting new scaling relationship for polythermal ice caps and ice sheets.

Similar to the energy balance equation, introducing basal sliding laws and other boundary equations may introduce new dimensionless parameters. These new dimensionless parameters could generate novel and important scaling relationships but cannot change the other dimensionless quantities presented above. In section 7, the derivation of the volume‐area scaling relationship will depend on a subset of the dimensionless parameters already derived in equations (25), (26), (27), (28), (29), (30), (31), (32), (33), (34), (35), (36), (37), (38), (39).

### 4.2 Directional Analysis

A Buckingham Pi analysis ensures dimensional compatibility, but makes no distinction between different orientations; nothing in the fundamental dimensions or dimensionless groups clearly distinguishes between horizontal and vertical directions, for example. This distinction can be made by an orientational analysis [*Siano*, 1985a,1985b], in which each variable with a fundamental unit of length is assigned an orientation, and relationships between these orientations create angles. Angles are dimensionless, so they do not appear in a traditional Buckingham analysis, but information about these angles can be introduced to the existing dimensionless parameters.

For example, the variable *x* has dimensions of length *L* and a coordinate direction or orientation 1*_x_* (a unit vector pointing in the *x* direction). Together, the dimensions and orientation of variable *x* can be represented as the vector *L*1*_x_.* Similarly, *y* has dimensions and orientation *L*1*_y_*. The product *xy* has dimensions of *L* ^2^ and orientation 1*_x_*1*_y_*. As in vector calculus, the orientation of a planar area (sometimes called a vector area) is defined by a vector perpendicular to the surface. So an area lying in the *xy* plane has orientation 1*_z_* pointing in the *z* direction. In other words, 1*_x_*1*_y_* = 1*_z_*. Generally, products of orientations are defined by a Klein group with products 1*_i_*1*_j_* = 1*_k_* for *i* ≠ *j*, identity 1*_i_*1*_i_* = 1, and inverse 1*_i_* ^*−*1^ = 1*_i_* [*Siano*, 1985a, 1985b].

Extending this idea to three dimensions, consider the volume *V*, with dimensions *L* ^3^ and orientation 1*_x_*1*_y_*1*_z_*. By the previous rules, 1*_x_*1*_y_*1*_z_* = (1*_x_*1*_y_*)1*_z_* = 1*_z_*1*_z_* = 1. Volume thus has no directional orientation, which makes intuitive sense. Glacier volume is sometimes defined as *V* = *Sh*, where *S* is surface area and *h* is a thickness perpendicular to *S*. A *dimensional* analysis would suggest that *V* expressed as area *S* times width *w* is equally valid (despite clearly being wrong) because both sides of this equation have the same dimensions of *L* ^3^. However *V* = *Sw* is not *directionally* consistent because *L* ^3^1*_x_*1*_y_*1*_z_* ≠ (*L* ^2^1*_x_*1*_y_*) (*L*1*_y_*) = *L* ^3^1*_x_*. When creating physically meaningful equations, we are not free to choose length variables arbitrarily, but instead must always choose lengths that are directionally consistent. Although this example is trivial, it will be seen that directional consistency will eliminate the need for considering glacier slope in a later derivation of glacier volume‐area scaling. However, it will also be seen that directional consistency will make the slope relevant when deriving the volume‐area scaling exponent for ice caps.

A directional analysis can be applied to each of the above dimensionless parameters Π_1_ to Π_15_. Considering equation (25), we can write the dimensions and orientation of Π_1_ = *x*/*y* as

(43) $$\frac{L1_{x}}{L1_{y}}=\frac{1_{x}}{1_{y}}=1_{x}1_{y}=1_{z}$$

This dimensionless parameter is evidently not directionally consistent. We can fix this by introducing a dimensionless angle in the *xy* plane. As with area, an angle has an orientation perpendicular to the plane in which it resides. An angle *θ*_*xy*_ lying in the *xy* plane has orientation 1*_z_*. Likewise, any odd power of *θ*_*xy*_ has orientation 1*_z_*. Therefore, we can modify equation (25) to read

(44) $$\Pi_{1}{\theta_{xy}}^{k}=\frac{x}{y}$$

This is both dimensionally and directionally consistent when *k* is an odd integer. Although the directional analysis provides no additional guidance, knowledge of the physics can further constrain the value of *k*. In this case, *k* = 1 is the obvious choice.

For some additional insight about the angle, note that the two variables *x* and *y* give magnitudes along the *x* and *y* axes. Therefore, *x* and *y* define the two perpendicular sides of a right triangle. The ratio of the sides of the triangle *x*/*y* defines the trigonometric tangent, tan *θ*, where *θ* is one of the angles of the triangle. The tangent has a series expansion with only odd powers of *θ*, which is consistent with the odd integer values of *k* in equation (44).

A similar analysis introduces angles to many of the other dimensionless parameters. For example,

(45) $$\Pi_{2}{\theta_{xz}}^{k}=\frac{x}{z}$$

for odd *k*, and physical intuition tells us that *θ*_*xz*_ will be the slope of the glacier when the *z* axis is parallel to gravity and the *x* axis is in the primary direction of flow. Equation (45) has been suggested in the simplified context of a block‐shaped glacier by *Harrison* [2013, equation (65)] (where Π_2_ and *zθ*_*xz*_ in this review are 1/(2 K) and $\tilde{H}$ in Harrison's analysis—combine Harrison's equations (37), (38), and (65)). The directional analysis confirms that Harrison's dimensional relationship is valid for all glaciers of any shape and not just parallel‐sided blocks.

Likewise,

(46) $$\Pi_{4}{\theta_{xz}}^{k}=\frac{u_{x}}{u_{z}}$$

where again the angle *θ*_*xz*_ is the same slope of the glacier. By inspection, Π_3_, Π_6_, and Π_7_ are modified in the same way. For example,

(47) $$\Pi_{6}{\theta_{xz}}^{k}=\frac{g_{x}}{g_{z}}$$

Note that stress *σ*_*ij*_ has orientation 1*_i_*1*_j_* (stress is force per unit area and has an orientation that is a product of the force vector orientation and the area vector orientation). Similarly, strain rate  ${\dot{\varepsilon }}_{ij}$ has orientation 1*_i_*1*_j_.* Therefore, via the constitutive equation, the rate parameter *A* has orientation 1_*i*_^*n* − 1^1_*j*_^*n* − 1^. It follows that each of Π_11_ through Π_15_ have an associated angle *θ*_*xy*_, *θ*_*xz*_, or *θ*_*yz*_. However, Π_5_, Π_8_, Π_9_, and Π_10_ are directionally consistent as written.

With directional consistency, the dimensionless parameter Π_9_ can be rewritten in a well‐known form that includes the glacier slope *θ*_*xz*_. From (33) and (47),

(48) $$\begin{array}{c} \Pi_{9}=\frac{A\rho^{n}{g_{x}}^{n}z^{n+1}}{u_{x}}\\ =\frac{A\rho^{n}{\left(\Pi_{6}g_{z}\theta_{xz}\right)}^{n}z^{n+1}}{u_{x}} \end{array}$$

Rearranging terms,

(49) $$u_{x}=\frac{{\Pi_{6}}^{n}}{\Pi_{9}}A{\left(\rho g_{z}\theta_{xz}\right)}^{n}z^{n+1}$$

This equation is used later when deriving the volume‐area exponent of an ice cap.

### 4.3 Discussion of Dimensionless Parameters

The fact that the Buckingham Pi Theorem can be applied with almost no knowledge of underlying processes is both its strength and its weakness. While the results embodied in the 15 dimensionless groups defined above were obtained with essentially no knowledge of glacier mechanics, neither can much understanding of the underlying mechanics be deduced from those 15 groups. For example, the derivation of (49) from a simple dimensional and directional analysis is remarkable, but its genesis in fundamental physics is completely unknown. However, the classic laminar flow derivation of a nearly identical result [e.g., *Cuffey and Paterson*, 2010] is laden with assumptions like plane strain and uniform slope, and the broad relevance of (49) is obscured. The Buckingham Pi methodology required no assumptions whatsoever beyond the choice of appropriate variables and their units. Nevertheless, caution and physical insight is still required. Dimensional analysis can incorrectly conflate two unrelated variables with the same dimensions, leading to physically implausible dimensionless groups. (Torque and energy are classic examples of dimensionally identical but nonequivalent variables, although they are distinguishable by their orientations, in the sense of *Siano* [1985a, 1985b]).

For creeping ice, the Buckingham Pi Theorem gives the correct dimensionless groups (no more and no less) without conflating dimensionally equivalent variables, but this can only be known with the benefit of hindsight and after significant experience with the relevant continuum equations. To dispel any uncertainties and to make each dimensionless parameter's genesis clearer, the following section describes an alternative to the Pi Theorem, a *stretching symmetry* analysis of the underlying differential equations. The advantage of stretching is its obvious glaciological and continuum mechanical underpinnings; the disadvantage is a long derivation, but we believe that in the context of this review, exactitude is both warranted and essential. Those who wish to skip the stretching symmetries can jump to section 6.

## 5 Stretching Analysis of Continuum Equations

Long‐time practitioners of fluid mechanics will recognize that ratios of each term in the mass, momentum, and constitutive equations give nondimensional relationships. The process of taking ratios is intuitive and straight forward in most cases, though complicated nonlinear terms, derivatives, and integrals can require physically intuited substitutions of the relevant scaled parameters. In these more complicated cases, taking ratios relies on assigning characteristic variables in a manner similar to a dimensional analysis and does not therefore have the advantage of being an independent technique with independent verification of the dimensionless groups derived above. On the other hand, the common practice of taking ratios of terms does yield many physical insights about the origins of each dimensionless group.

Taking ratios of terms to derive dimensionless groups is a learned procedure rather than a mathematically justified proof of the resulting groups. Likewise, many theoreticians can write the relevant dimensionless parameters “on inspection” of the equations, but this learned intuition does little to help newcomers to glaciology. A mathematically rigorous technique is preferable, especially given the occasional misconceptions about the mathematical origins of volume‐area scaling in glaciology (see Table 1). Those who are looking to gain additional insights or who are concerned about rigor may find that the following *method of stretchings* [e.g., *Logan*, 2004] remove some of the ambiguities presented by shortcuts such as taking ratios. However, those glaciologists comfortable with taking ratios or with writing the relevant dimensionless quantities on inspection may safely skip section 5.

### 5.1 The Stretching Transform and Continuity Equation

We start by performing a stretching transformation on the integrated three‐dimensional continuity equation (13). Each variable *X* in the continuity equation will be multiplied by a quantity that represents the rescaling of that variable following a switch to a new unit of measurement. In particular, we multiply each variable by the scalar factor $\lambda^{k_{X}}$, where *λ* is the same arbitrary fixed constant for all variables and *k_X_* is a constant stretching exponent specific to the variable *X*. Let a bar indicate a rescaled quantity. Then for each variable in (13) and (14) the stretching transforms are

(50) $$\begin{array}{l} \begin{array}{ccc} \bar{h}=\lambda^{k_{h}}h & {\bar{h}}_{s}=\lambda^{k_{h_{s}}}h_{s} & {\bar{h}}_{b}=\lambda^{k_{h_{b}}}h_{b}\\ \bar{x}=\lambda^{k_{x}}x & \bar{y}=\lambda^{k_{y}}y & \bar{z}=\lambda^{k_{z}}z\\ {\bar{u}}_{x}=\lambda^{k_{u_{x}}}u_{x} & {\bar{u}}_{y}=\lambda^{k_{u_{y}}}u_{y} & \dot{b}–=\lambda^{k_{b}}\dot{b} \end{array}\\ \;\bar{t}=\lambda^{k_{t}}t \end{array}$$

An appropriate stretching of each variable must preserve the original continuity equations (13) and (14). For example, the rescaled and equivalent version of (14) is

(51) $$\bar{h}={\bar{h}}_{s}-{\bar{h}}_{b}$$

This can be directly related to the original equation by substituting the rescaled quantities from (50) into (51).

(52) $$\lambda^{k_{h}}h=\lambda^{k_{h_{s}}}h_{s}-\lambda^{k_{h_{b}}}h_{b}$$

Note that the rescaling (52) preserves the original equation (14) if and only if the constants can be factored out, in which case

(53) $$\lambda^{k_{h}}=\lambda^{k_{h_{s}}}=\lambda^{k_{h_{b}}}$$

Or equivalently,

(54) $$k_{h}=k_{h_{s}}=k_{h_{b}}$$

This places constraints on the variables *h*, *h_s_*, and *h_b_*. Not unexpectedly, equation (54) indicates that these topographic variables must always be rescaled by the same amount. For example, if the thickness *h* is stretched by a factor of $\lambda^{k_{h}}=2$, then *h_s_* and *h_b_* must also be rescaled by the exact same factor of 2.

Each term in the continuity equation (13) is handled in a similar manner. For the first term,

(55) $$\begin{array}{c} \frac{\partial \bar{h}}{\partial \bar{t}}=\frac{\partial }{\partial \bar{t}}\left(\lambda^{k_{h}}h\right)\\ =\lambda^{k_{h}}\frac{\partial h}{\partial \bar{t}}\\ =\lambda^{k_{h}}\frac{\partial h}{\partial t}\;\frac{\partial t}{\partial \bar{t}}\\ =\lambda^{k_{h}}\frac{\partial h}{\partial t}\lambda^{-k_{t}}\\ =\lambda^{k_{h}-k_{t}}\frac{\partial h}{\partial t} \end{array}$$

The transform of a derivative appears as the subtraction of the exponents associated with the variables of differentiation.

Similarly, integration is represented by addition of stretching constants. For the third term of the continuity equation (13),

(56) $$\begin{array}{c} \frac{\partial }{\partial \bar{x}}\int_{{\bar{h}}_{b}}^{{\bar{h}}_{s}}{\bar{u}}_{x}d\bar{z}=\lambda^{-k_{x}}\frac{\partial }{\partial x}\int_{\bar{z}={\bar{h}}_{b}}^{\bar{z}={\bar{h}}_{s}}\lambda^{k_{u_{x}}}u_{x}\frac{d\bar{z}}{dz}\;\frac{dz}{d\bar{z}}\;d\bar{z}\\ =\lambda^{-k_{x}}\frac{\partial }{\partial x}\int_{z=\lambda^{-k_{z}}{\bar{h}}_{b}}^{z=\lambda^{-k_{z}}{\bar{h}}_{s}}\lambda^{k_{u_{x}}}u_{x}\;\lambda^{k_{z}}\frac{dz}{d\bar{z}}\;d\bar{z}\\ =\lambda^{k_{u_{x}}-k_{x}}\frac{\partial }{\partial x}\int_{z=\lambda^{k_{h}-k_{z}}h_{b}}^{z=\lambda^{k_{h}-k_{z}}h_{s}}u_{x}\;\lambda^{k_{z}}dz\\ =\lambda^{k_{u_{x}}-k_{x}}\frac{\partial }{\partial x}\int_{z=\lambda^{k_{h}-k_{h}}h_{b}}^{z=\lambda^{k_{h}-k_{h}}h_{s}}u_{x}\;\lambda^{k_{h}}dz\\ =\lambda^{k_{u_{x}}-k_{x}+k_{h}}\left(\frac{\partial }{\partial x}\int_{h_{b}}^{h_{s}}u_{x}dz\right) \end{array}$$

The stretching constant *k_z_* is not part of equation (56) because *z* must scale identically to the limits of integration (the limits replace *z* in the evaluation of the definite integral). Thus, *z* scales identically to both *h_s_* and *h_b_* which from (54) all scale identically to *h*

(57) $$k_{h}=k_{h_{s}}=k_{h_{b}}=k_{z}$$

where *z* is a dummy variable that plays no role in the final solution and can be ignored.

Applying similar arguments to the remaining terms in the continuity equation (13) gives

(58) $$\lambda^{k_{h}-k_{t}}\frac{\partial h}{\partial t}=\lambda^{k_{b}}\overset{\text{⋅}}{b}-\lambda^{k_{u_{x}}-k_{x}+k_{h}}\left(\frac{\partial }{\partial x}\int_{h_{b}}^{h_{s}}u_{x}dz\right)-\lambda^{k_{u_{y}}-k_{y}+k_{h}}\left(\frac{\partial }{\partial y}\int_{h_{b}}^{h_{s}}u_{y}dz\right)$$

As demonstrated in equation (53), this stretched form of the continuity equation is equivalent to the original continuity equation (13) if and only if the terms with *λ* can be factored out. This is only the case when

(59) $$\begin{array}{c} \lambda^{k_{h}-k_{t}}=\lambda^{k_{b}}\\ =\lambda^{k_{u_{x}}-k_{x}+k_{h}}\\ =\lambda^{k_{u_{y}}-k_{y}+k_{h}} \end{array}$$

or equivalently

(60) $$\begin{array}{c} k_{h}-k_{t}=k_{b}\\ =k_{u_{x}}-k_{x}+k_{h}\\ =k_{u_{y}}-k_{y}+k_{h} \end{array}$$

The solution of equations (57) and (60) gives the full set of stretching exponents imposed by the continuity equation. These constraints dictate how the variables of the continuity equation must change with respect to each other. For example, if the thickness is rescaled by a factor of $\lambda^{k_{h}}$, then the first line of (60) shows that the time and balance rate must also be rescaled such that *k*_*h*_ = *k*_*b*_ + *k*_*t*_.

As a final step, each of the constraints can be rendered as dimensionless numbers. By rearranging terms in the stretching transforms (50),

(61) $$\begin{array}{l} \begin{array}{c} \begin{array}{cc} \;\lambda^{k_{h}}=\bar{h}/h & \lambda^{k_{t}}=\bar{t}/t\; \end{array}\\ \begin{array}{cc} \;\lambda^{k_{x}}=\bar{x}/x & \lambda^{k_{b}}=\bar{\dot{b}}/\dot{b}\; \end{array}\\ \begin{array}{cc} \;\lambda^{k_{y}}=\bar{y}/y & \lambda^{k_{u_{x}}}={\bar{u}}_{x}/u_{x} \end{array} \end{array}\\ \lambda^{k_{u_{y}}}={\bar{u}}_{y}/u_{y}\; \end{array}$$

Substitution into (59) gives

(62) $$\begin{array}{c} \left(\frac{\bar{h}}{h}\right)\left(\frac{t}{\bar{t}}\right)=\left(\frac{\bar{\dot{b}}}{\dot{b}}\right)\\ =\left(\frac{{\bar{u}}_{x}}{u_{x}}\right)\left(\frac{\bar{h}}{h}\right)\left(\frac{x}{\bar{x}}\right)\\ =\left(\frac{{\bar{u}}_{y}}{u_{y}}\right)\left(\frac{\bar{h}}{h}\right)\left(\frac{y}{\bar{y}}\right) \end{array}$$

Six pairings of ratios can be formed from these three equations, each giving a unique nondimensional number. For example, consider the first line of equation (62). Separating stretched from unstretched variables gives

(63) $$\frac{t\dot{b}}{h}=\frac{\bar{t}\;\bar{\dot{b}}}{\bar{h}}$$

By construction, each side of the equation is dimensionless and has a magnitude that depends on the values selected (or measured) for each variable.

Let Π_*c*1_ be the nondimensional number represented by equation (63), where the subscript *c* indicates that it was derived from continuity. Then,

(64) $$\Pi_{c1}=\frac{t\dot{b}}{h}$$

This dimensionless group is closely related to the well‐known relationship characterizing glacier response time as conceived by *Jóhannesson et al*. [1989]. Similarly, the left‐hand side of (62) and the second line of (62) can be rearranged to give

(65) $$\Pi_{c2}=\frac{t\;u_{x}}{x}$$

The left‐hand side of (62) and the third line of (62) can be rearranged to give

(66) $$\Pi_{c3}=\frac{t\;u_{y}}{y}$$

With the three separate equalities in (62), there are six possible combinations of terms. The three others are

(67) $$\Pi_{c4}=\frac{\dot{b}\;x}{u_{x}h}$$

(68) $$\Pi_{c5}=\frac{\dot{b}\;y}{u_{y}h}$$

and

(69) $$\Pi_{c6}=\frac{x\;u_{y}}{y\;u_{x}}$$

Additionally, three trivial relationships can be derived from (54).

(70) $$\Pi_{c7}=\frac{h}{h_{s}}$$

(71) $$\Pi_{c8}=\frac{h}{h_{b}}$$

(72) $$\Pi_{c9}=\frac{h_{s}}{h_{b}}$$

Note again that *z* never appears outside the integrand in (13) and is irrelevant to any final solution.

#### 5.1.1 Uniqueness and Relationship to Buckingham Pi

In general, the stretching symmetries do not guarantee a set of unique relationships. While the redundancies are usually eliminated, there is no harm in their retention. In particular,

(73) $$\Pi_{c4}=\frac{\Pi_{c1}}{\Pi_{c2}}$$

(74) $$\Pi_{c5}=\frac{\Pi_{c1}}{\Pi_{c3}}$$

(75) $$\Pi_{c6}=\frac{\Pi_{c3}}{\Pi_{c2}}$$

and

(76) $$\Pi_{c9}=\frac{\Pi_{c8}}{\Pi_{c7}}$$

Furthermore, *h = h_s_* − *h_b_* (see equation (14)), so Π_*c*8_ is not independent of Π_*c*7_, and

(77) $$\Pi_{c8}=\frac{\Pi_{c7}}{1-\Pi_{c7}}$$

Thus, only Π_*c*1_, Π_*c*2_, Π_*c*3_, and Π_*c*7_ are unique dimensionless parameters. All others can be constructed from these four.

A different uniqueness problem can arise in a naïve application of the Buckingham Pi Theorem. Without prior knowledge of the continuity equation, one might hypothesize that there are eight independent and relevant variables ($x,y,h,h_{s},t,\overset{\text{˙}}{b},u_{x},u_{y}$). These variables depend on only two fundamental dimensions, length *L* and time *T*. Therefore, there will be 8 − 2 = 6 possible dimensionless parameters from which all others can be constructed. These are two more parameters than would be derived with a priori knowledge of the continuity equation. Following a standard Buckingham method [e.g., *Welty et al*., 1984], the dimensionless parameters are Π_*c*1_, Π_*c*2_, Π_*c*3_, and Π_*c*7_, as before, but the Buckingham method also includes the extra geometric ratios *x*/*h* and *y*/*h* that do not arise from continuity alone. Combining these extra ratios with Π_*c*1_, Π_*c*2_, and Π_*c*3_ gives additional kinematic ratios such as *u_x_*/*u_y_*; again, the kinematic ratios do not arise from the continuity equation.

It will be seen that these geometric and kinematic ratios will be obtained later from the constitutive equation. So while the Buckingham Pi Theorem provides two extra and seemingly extraneous dimensionless parameters with respect to continuity alone, these parameters are still fundamental and not extraneous to the overall problem of glacier flow. Because the Buckingham Pi Theorem does not depend on a priori knowledge of the underlying equations, it does not divide the variables' contributions according to their particular roles in separate equations (e.g., their role in the continuity equation versus the constitutive equation versus the equations of motion). This is another source of both strength and weakness in the Buckingham Pi analysis: while significant progress can be made without a priori knowledge of the governing equations, it is not possible to trace elements of the analysis back to the principles (represented by continuum equations) from which they originate.

#### 5.1.2 Alternate and Generalized Derivation

The results of the last section were obtained from the integral form of the continuity equation (13), which explicitly includes the glacier thickness. Alternatively, the differential form of continuity (12) can be used. Thickness and mass balance rate are replaced by the vertical dimension *z* and vertical velocity *u_z_* and each appears explicitly in the resulting dimensionless parameters. For incompressible ice, ∂*ρ*/∂*t* on the left‐hand side of (12) might appear negligible. However, this term cannot be ignored because abrupt changes in density define the domain boundaries, in particular the surface and the bed of the glacier where mass can be added or removed (snowfall, melting, etc.). In fact, in the vertically integrated form of continuity (13), the term ∂*h*/∂*t* comes out of the vertical integral of ∂*ρ*/∂*t* after an application of the Leibnitz integral rule.

Using the same stretching transformation described above,

(78) $$\begin{array}{c} \lambda^{k_{\rho }-k_{t}}\frac{\partial \rho }{\partial t}=-\lambda^{k_{\rho }+k_{u_{x}}-k_{x}}\frac{\partial }{\partial x}\;\left(\rho \;u_{x}\right)-\lambda^{k_{\rho }+k_{u_{y}}-k_{y}}\frac{\partial }{\partial y}\left(\rho \;u_{y}\right)\\ -\lambda^{k_{\rho }+k_{u_{z}}-k_{z}}\frac{\partial }{\partial z}\;\left(\rho \;u_{z}\right)-\lambda^{k_{\mu }}\dot{\mu } \end{array}$$

and

(79) $$\begin{array}{c} \left(\frac{\bar{\rho }}{\rho }\right)\left(\frac{t}{\bar{t}}\right)=\left(\frac{\bar{\rho }}{\rho }\right)\left(\frac{{\bar{u}}_{x}}{u_{x}}\right)\left(\frac{x}{\bar{x}}\right)\\ =\left(\frac{\bar{\rho }}{\rho }\right)\left(\frac{{\bar{u}}_{y}}{u_{y}}\right)\left(\frac{y}{\bar{y}}\right)\\ =\left(\frac{\bar{\rho }}{\rho }\right)\left(\frac{{\bar{u}}_{z}}{u_{z}}\right)\left(\frac{z}{\bar{z}}\right)\\ =\left(\frac{\bar{\dot{\mu }}}{\dot{\mu }}\right) \end{array}$$

Solving for the unstretched parameters gives three nondimensional relationships of the form

(80) $$\Pi_{c}=\frac{t\;u_{i}}{x_{i}}$$

where *i = x, y, z* and gives an additional source term

(81) $$\begin{array}{c} \Pi_{c\dot{\mu }}=\frac{t\dot{\mu }}{\rho }\\ =\frac{t\dot{\mu }}{\rho }\left(\frac{x_{i}}{x_{i}}\right) \end{array}$$

We multiply by (*x*_*i*_/*x*_*i*_) to show that if density is constant, then $x_{i}\dot{\mu }/\rho$ is a measure of length per unit time or velocity, and therefore (81) is equivalent to the other parameters in (80). In other words, for constant density ice, $\Pi_{c\dot{\mu }}$ is redundant and only equation Π_*c*_ is necessary.

Fundamentally, Π*_c_* is the same as Π_*c*1_, Π_*c*2_, and Π_*c*3_ (equations (64), (65), (66)), previously derived from the integrated form of continuity (13). To equate the two sets of dimensionless parameters, the characteristic value of *z* is simply selected to be the characteristic thickness. Likewise, the characteristic vertical velocity is selected as the characteristic mass balance rate. Equation (80) is more general, but equations (64), (65), (66) are more widely used, such as in numerical models based on integrated forms of continuity. Either set can be used.

### 5.2 Stretching Analysis of the Equations of Motion

Dimensionless parameters associated with the equations of motion (15) are derived by the same process as outlined for the continuity equation above. Note that we do not use the simpler stress equilibrium equations because the material derivative (16) is relevant when integrated. As with the continuity equation, an integral of (15) can make the time rate of change D/D*t* nonzero at nonstationary boundaries. For example, integrating (15) over depth and width gives the rate of change of flux through a glacier cross section; such temporal changes in flux have been observed on glaciers and can be significant [*O'Neel et al*., 2005]. If the material derivative is ignored, the stretching symmetries for glacier flow will yield one fewer dimensionless parameters than predicted by the Buckingham Pi Theorem. That contradiction would imply that either the continuum mechanical description is incomplete or that the Buckingham method has considered too many variables or too many fundamental dimensions. The list of variables and dimensions (mass, length, and time) are reasonable, so we would correctly conclude that the material derivative is essential to the continuum mechanics of ice.

Each variable in the equations of motion (15) is rescaled such that

(82) $$\begin{array}{cc} {\bar{x}}_{i}=\lambda^{k_{x_{i}}} & {\bar{\sigma }}_{ij}=\lambda^{k_{\sigma_{ij}}}\sigma_{ij}\\ {\bar{u}}_{{}_{i}}=\lambda^{k_{u_{i}}}u_{i} & {\bar{g}}_{i}=\lambda^{k_{g_{i}}}g_{i}\\ \bar{\rho }=\lambda^{k_{\rho }}\rho & \bar{t}=\lambda^{k_{t}}t \end{array}$$

The rescaled equations become

(83) $$\frac{\partial \;{\bar{\sigma }}_{ii}}{\partial \;{\bar{x}}_{i}}+\frac{\partial \;{\bar{\sigma }}_{ij}}{\partial \;{\bar{x}}_{j}}+\frac{\partial \;{\bar{\sigma }}_{ik}}{\partial \;{\bar{x}}_{k}}+\bar{\rho }\;{\bar{g}}_{i}=\bar{\rho }\left(\frac{\partial \;{\bar{u}}_{i}}{\partial \;\bar{t}}+{\bar{u}}_{x}\frac{\partial \;{\bar{u}}_{i}}{\partial \;\bar{x}}+{\bar{u}}_{y}\frac{\partial \;{\bar{u}}_{i}}{\partial \;\bar{y}}+{\bar{u}}_{z}\frac{\partial \;{\bar{u}}_{i}}{\partial \;\bar{z}}\right)$$

Substituting (82) into (83),

(84) $$\begin{array}{l} \lambda^{k_{\sigma_{ii}}-k_{x_{i}}}\frac{\partial \sigma_{ii}}{\partial x_{i}}+\lambda^{k_{\sigma_{ij}}-k_{x_{j}}}\frac{\partial \sigma_{ij}}{\partial x_{j}}+\lambda^{k_{\sigma_{ik}}-k_{x_{k}}}\frac{\partial \sigma_{ik}}{\partial x_{k}}+\lambda^{k_{\rho }+k_{g_{i}}}\rho g_{i}\\ \;=\lambda^{k_{\rho }+k_{u_{i}}-k_{t}}\rho \frac{\partial u_{i}}{\partial t}+\lambda^{k_{\rho }+k_{u_{x}}+k_{u_{i}}-k_{x}}\rho u_{x}\frac{\partial u_{i}}{\partial x}\\ \;+\lambda^{k_{\rho }+k_{u_{y}}+k_{u_{i}}-k_{y}}\rho u_{y}\frac{\partial u_{i}}{\partial y}+\lambda^{k_{\rho }+k_{u_{z}}+k_{u_{i}}-k_{z}}\rho u_{z}\frac{\partial u_{i}}{\partial z} \end{array}$$

This matches the original equations of motion (15) if and only if the terms in *λ* factor out, i.e.,

(85) $$\begin{array}{c} \lambda^{k_{\sigma_{ii}}-k_{x_{i}}}=\lambda^{k_{\sigma_{ij}}-k_{x_{j}}}\\ =\lambda^{k_{\sigma_{ik}}-k_{x_{k}}}\\ =\lambda^{k_{\rho }+k_{g_{i}}}\\ =\lambda^{k_{\rho }+k_{u_{i}}-k_{t}}\\ =\lambda^{k_{\rho }+k_{u_{x}}+k_{u_{i}}-k_{x}}\\ =\lambda^{k_{\rho }+k_{u_{y}}+k_{u_{i}}-k_{y}}\\ =\lambda^{k_{\rho }+k_{u_{z}}+k_{u_{i}}-k_{z}} \end{array}$$

By substituting (82) into (85),

(86) $$\begin{array}{c} \left(\frac{{\bar{\sigma }}_{ii}}{\sigma_{ii}}\right)\left(\frac{x_{i}}{{\bar{x}}_{i}}\right)=\left(\frac{{\bar{\sigma }}_{ij}}{\sigma_{ij}}\right)\left(\frac{x_{j}}{{\bar{x}}_{j}}\right)\\ =\left(\frac{{\bar{\sigma }}_{ik}}{\sigma_{ik}}\right)\left(\frac{x_{k}}{{\bar{x}}_{k}}\right)\\ =\left(\frac{\bar{\rho }}{\rho }\right)\left(\frac{{\bar{g}}_{i}}{g_{i}}\right)\\ =\left(\frac{\bar{\rho }}{\rho }\right)\left(\frac{{\bar{u}}_{i}}{u_{i}}\right)\left(\frac{t}{\bar{t}}\right)\\ =\left(\frac{\bar{\rho }}{\rho }\right)\left(\frac{{\bar{u}}_{l}}{u_{l}}\right)\left(\frac{{\bar{u}}_{i}}{u_{i}}\right)\left(\frac{x_{l}}{{\bar{x}}_{l}}\right) \end{array}$$

where *i*, *j*, *k*, *l* ∈ {*x*, *y*, *z*}, so each line in (86) represents more than one equality. Each pair of equalities in the expanded (86) gives one dimensionless parameter. As with the continuity equation, some parameters are not unique and can be derived from others. After eliminating redundancies,

(87) $$\Pi_{\sigma 1}=\left(\frac{x_{i}}{x_{j}}\right)\left(\frac{\sigma_{ij}}{\sigma_{jk}}\right)$$

(88) $$\Pi_{\sigma 2}=\frac{\rho g_{i}x_{j}}{\sigma_{ij}}$$

(89) $$\Pi_{\sigma 3}=\frac{t\;g_{i}}{u_{i}}$$

(90) $$\Pi_{\sigma 4}=\frac{t\;u_{i}}{x_{i}}$$

where *i*, *j*, *k* ∈ {*x*, *y*, *z*}. The subscript *σ* on the left‐hand side indicates that the dimensionless number has been derived from the equations of motion.

Note that combinations of Π_*σ*1_ and Π_*σ*2_ give ratios of the form

(91) $$\Pi_{\sigma 5}=\left(\frac{x_{i}}{x_{j}}\right)\left(\frac{g_{j}}{g_{i}}\right)$$

Geometric similarity is derived in the next section from the constitutive relationship, so equation (91) will imply that gravity scales identically in all directions, as expected.

Interestingly, equation (90) makes the dimensionless parameters from the continuity equation superfluous. This is a direct consequence of the material derivative, which has the same dimensional structure as the continuity equation: compare the dimensions of (12), (16). The statement of continuity is helpful for our intuition but unnecessary for a dimensional analysis. This connection between dimensionless parameters derived from the material derivative and from continuity could not have been inferred from the Buckingham Pi method.

### 5.3 Stretching Analysis of the Constitutive Equation

The nonlinearity of the flow law makes a stretching analysis more complicated but follows the same procedure outlined above. Expanding the summations and substituting the definitions of deviatoric stress (18) and strain rate (19) into the flow law gives

(92) $$\begin{array}{l} {\dot{\varepsilon }}_{ij}=A\left[\frac{1}{2}{\left(\sigma_{xx}-\frac{1}{3}\left(\sigma_{xx}+\sigma_{yy}+\sigma_{zz}\right)\right)}^{2}\right.\\ \;+\frac{1}{2}{\left(\sigma_{yy}-\frac{1}{3}\left(\sigma_{xx}+\sigma_{yy}+\sigma_{zz}\right)\right)}^{2}\\ \;+\frac{1}{2}{\left(\sigma_{zz}-\frac{1}{3}\left(\sigma_{xx}+\sigma_{yy}+\sigma_{zz}\right)\right)}^{2}\\ \;+{\left.{\sigma_{xy}}^{2}+{\sigma_{xz}}^{2}+{\sigma_{yz}}^{2}\right]}^{\frac{n-1}{2}}\sigma_{ij}^{\prime } \end{array}$$

After a further expansion of terms and significant manipulation, this reduces to

(93) $$\begin{array}{l} {\dot{\varepsilon }}_{ij}=A\left[\frac{1}{3}\;{\sigma_{xx}}^{2}\right.+\frac{1}{3}{\sigma_{yy}}^{2}+\frac{1}{3}\;{\sigma_{zz}}^{2}\\ \;-\frac{1}{3}\sigma_{xx}\sigma_{yy}-\frac{1}{3}\sigma_{xx}\sigma_{zz}-\frac{1}{3}\;\sigma_{yy}\sigma_{zz}\\ \;+{\left.{\sigma_{xy}}^{2}+{\sigma_{yz}}^{2}+{\sigma_{xz}}^{2}\right]}^{\frac{n-1}{2}}\sigma_{ij}^{\prime } \end{array}$$

To simplify a very long presentation, we temporarily use *n* = 3, a widely accepted value supported by both data and dislocation theory [e.g., *Hooke*, 2005; *Cuffey and Paterson*, 2010]. This should not be construed as an assumption. By inspection, the more general expansion will follow in an obvious manner (as presented shortly), but for those who are so inclined, the full expansion can be derived using a Taylor series or noninteger generalization of the Binomial Theorem.

For *n* = 3 and *i* ≠ *j*, the deviatoric stress $\sigma_{ij}^{\prime }$ in (93) reduces to the full shear stress *σ*_*ij*_. Multiplying term by term gives

(94) $$\begin{array}{c} {\dot{\varepsilon }}_{ij}=\frac{1}{2}\left(\frac{\partial u_{i}}{\partial x_{j}}+\frac{\partial u_{j}}{\partial x_{i}}\right)\\ \;=A\left(\frac{1}{3}\;{\sigma_{xx}}^{2}\sigma_{ij}\right.+\frac{1}{3}{\sigma_{yy}}^{2}\sigma_{ij}+\frac{1}{3}\;{\sigma_{zz}}^{2}\sigma_{ij}\\ \;-\frac{1}{3}\sigma_{xx}\sigma_{yy}\sigma_{ij}-\frac{1}{3}\sigma_{xx}\sigma_{zz}\sigma_{ij}-\frac{1}{3}\;\sigma_{yy}\sigma_{zz}\sigma_{ij}\\ \;+{\sigma_{xy}}^{2}\sigma_{ij}+{\sigma_{yz}}^{2}\sigma_{ij}+\left.{\sigma_{xz}}^{2}\sigma_{ij}\right) \end{array}$$

For *n* = 3 and *i* = *j*, the deviatoric stress $\sigma_{ij}^{\prime }$ in (93) is written in terms of its full‐stress equivalent per (18). Expanding term by term, and after some manipulation,

(95) $$\begin{array}{c} {\dot{\varepsilon }}_{ii}=\frac{\partial u_{i}}{\partial x_{i}}\\ =A\left(\frac{1}{3}\;{\sigma_{xx}}^{2}\sigma_{ii}\right.+\frac{1}{3}{\sigma_{yy}}^{2}\sigma_{ii}+\frac{1}{3}\;{\sigma_{zz}}^{2}\sigma_{ii}\\ \;-\frac{1}{3}\sigma_{xx}\sigma_{yy}\sigma_{ii}-\frac{1}{3}\sigma_{xx}\sigma_{zz}\sigma_{ii}-\frac{1}{3}\sigma_{yy}\sigma_{zz}\sigma_{ii}+\frac{1}{3}\;\sigma_{xx}\sigma_{yy}\sigma_{zz}\\ +{\sigma_{xy}}^{2}\sigma_{ii}+{\sigma_{yz}}^{2}\sigma_{ii}+{\sigma_{xz}}^{2}\sigma_{ii}\\ -\frac{1}{9}{\sigma_{xx}}^{3}-\frac{1}{9}{\sigma_{yy}}^{3}-\frac{1}{9}\;{\sigma_{zz}}^{3}\\ -\frac{1}{3}{\sigma_{xy}}^{2}\sigma_{xx}-\frac{1}{3}{\sigma_{yz}}^{2}\sigma_{xx}-\frac{1}{3}\;{\sigma_{xz}}^{2}\sigma_{xx}\\ -\frac{1}{3}{\sigma_{xy}}^{2}\sigma_{yy}-\frac{1}{3}{\sigma_{yz}}^{2}\sigma_{yy}-\frac{1}{3}\;{\sigma_{xz}}^{2}\sigma_{yy}\\ -\frac{1}{3}{\sigma_{xy}}^{2}\sigma_{zz}-\frac{1}{3}\;{\sigma_{yz}}^{2}\sigma_{zz}\left.-\frac{1}{3}\;{\sigma_{xz}}^{2}\sigma_{zz}\right) \end{array}$$

After stretching each variable as in previous sections, equations (94) and (95) give a set of equalities between rescaled and unscaled variables. From (94),

(96) $$\begin{array}{c} \left(\frac{A}{\bar{A}}\right)\left(\frac{{\bar{u}}_{i}}{u_{i}}\right)\left(\frac{x_{j}}{{\bar{x}}_{j}}\right)=\left(\frac{A}{\bar{A}}\right)\left(\frac{{\bar{u}}_{j}}{u_{j}}\right)\left(\frac{x_{i}}{{\bar{x}}_{i}}\right)\\ =\left(\frac{{\bar{\sigma }}_{kk}}{\sigma_{kk}}\right)\left(\frac{{\bar{\sigma }}_{ll}}{\sigma_{ll}}\right)\left(\frac{{\bar{\sigma }}_{ij}}{\sigma_{ij}}\right)\\ ={\left(\frac{{\bar{\sigma }}_{mp}}{\sigma_{mp}}\right)}^{2}\left(\frac{{\bar{\sigma }}_{ij}}{\sigma_{ij}}\right) \end{array}$$

where *i*, *j*, *k*, *l*, *m*, *p* ∈ {*x*, *y*, *z*}. From (95),

(97) $$\begin{array}{c} \left(\frac{A}{\bar{A}}\right)\left(\frac{{\bar{u}}_{i}}{u_{i}}\right)\left(\frac{x_{i}}{{\bar{x}}_{i}}\right)=\left(\frac{{\bar{\sigma }}_{jj}}{\sigma_{jj}}\right)\left(\frac{{\bar{\sigma }}_{kk}}{\sigma_{kk}}\right)\left(\frac{{\bar{\sigma }}_{ii}}{\sigma_{ii}}\right)\\ ={\left(\frac{{\bar{\sigma }}_{lm}}{\sigma_{lm}}\right)}^{2}\left(\frac{{\bar{\sigma }}_{ii}}{\sigma_{ii}}\right)\\ ={\left(\frac{{\bar{\sigma }}_{pp}}{\sigma_{pp}}\right)}^{3}\\ ={\left(\frac{{\bar{\sigma }}_{qr}}{\sigma_{qr}}\right)}^{2}\left(\frac{{\bar{\sigma }}_{ss}}{\sigma_{ss}}\right) \end{array}$$

where *i*, *j*, *k*, *l*, *m*, *p*, *q*, *r*, *s* ∈ {*x*, *y*, *z*}. As above, each pair of equalities in the expanded expressions produces one dimensionless parameter, giving a total of 55 dimensionless parameters from equation (96) and 231 from equation (97). As in previous sections, the vast majority of these parameters are not unique and can be derived from each other. To help reduce the large number of parameters, note that most pairs of terms involve only stresses (no velocities). Each of these gives a dimensionless parameter of the form

(98) $$\Pi_{G1}=\frac{\sigma_{ij}}{\sigma_{jk}}$$

where the subscript *G* indicates that the dimensionless parameter was derived from Glen's flow law. Note that none, some, or all of *i*, *j*, and *k* may be identical. In other words, every stress component must scale identically with every other stress component (both shear and normal). This is a statement of static similarity (i.e., similarity of forces in static equilibrium).

Other terms in (96) and (97) involve a velocity. Those pairs that include both a velocity and a stress will take the form

(99) $$\Pi_{G2}=\frac{u_{i}}{Ax_{j}{\sigma_{ij}}^{3}}$$

All other dimensionless parameters derived from (96) and (97) are nonunique and follow from combinations of Π_*G*1_ and Π_*G*2_.

Note that geometric similarity and kinematic similarity are easily derived from (98) and (99). Although not unique, these are important enough to write separately.

(100) $$\Pi_{G3}=\frac{x_{i}}{x_{j}}$$

(101) $$\Pi_{G4}=\frac{u_{i}}{u_{j}}$$

Through these terms the constitutive relationship implies geometric, kinematic, and static similarity. If the metric describing a glacier's geometry is stretched in one direction, then it must be stretched by the same amount in all other directions. If a velocity is scaled in one direction, then all other velocities must be scaled by the same amount. If forces are scaled in one direction, then forces must be scaled by the same amount in all other directions.

Generalizations from *n* = 3 to arbitrary *n* are now obvious. Any expansion of the right‐hand side of the constitutive equation (17) will contain terms with multiple and different stress components (see (94) and (95) for an example when *n* = 3). Static similarity (98) follows by default because each stress component will have to scale like all others. Each term in the expansion can have many different stress components, but their combined exponent will always be *n*. By replacing each stress component with the same stress component (static similarity), all terms in the expansion will have a single stress component with exponent *n*. Therefore, equation (99) is generalized to

(102) $$\Pi_{G5}=\frac{u_{i}}{Ax_{j}{\sigma_{ij}}^{n}}$$

## 6 Using Dimensionless Parameters

### 6.1 Summary of Dimensionless Parameters by Physical Origin

By combining all of the dimensionless parameters derived from stretching transformations of the continuity equation, the equations of motion, and the constitutive relationship, we arrive at the same set of 15 dimensionless numbers Π_1_ through Π_15_ that were derived from the Buckingham method. These can now be grouped by their physical origins.

Geometric similarity follows from the constitutive equations:

(103) $${\Pi_{1}}^{\prime }=\frac{x_{i}}{x_{j}}$$

Kinematic similarity follows from the constitutive equation:

(104) $${\Pi_{2}}^{\prime }=\frac{u_{i}}{u_{j}}$$

Static similarity follows from the constitutive equation:

(105) $${\Pi_{3}}^{\prime }=\frac{\sigma_{ij}}{\sigma_{jk}}$$

Gravitational similarity follows from the equations of motion when combined with geometric similarity:

(106) $${\Pi_{4}}^{\prime }=\frac{g_{i}}{g_{j}}$$

A statement about dynamics and gravity follows from the material derivative in the equations of motion:

(107) $${\Pi_{5}}^{\prime }=\frac{t^{2}g_{i}}{x_{i}}$$

A well‐known response time relationship follows from either the continuity equation or from the material derivative in the equations of motion (both lead to the same result):

(108) $${\Pi_{6}}^{\prime }=\frac{t\;u_{i}}{x_{i}}$$

A well‐known stress relationship follows from the equations of motion:

(109) $${\Pi_{7}}^{\prime }=\frac{x_{i}\rho g_{j}}{\sigma_{ij}}$$

Finally, a well‐known velocity relationship follows from the constitutive relationship combined with the equations of motion (combining (102) with (109)):

(110) $${\Pi_{8}}^{\prime }=\frac{A\rho^{n}{g_{i}}^{n}{x_{j}}^{n+1}}{u_{i}}$$

Expansion of the *ij* notation gives all 15 parameters. We use a prime to distinguish these from the same parameters derived by the Buckingham Pi method in section 4.

Despite its origin in the material derivative, the (*x*/*t* ^2^)^−1^ in equation (107) does not have to be interpreted as an acceleration of ice. Instead, it is a fundamental statement about the way gravity must be rescaled if we change the size of a glacier but do not want the dynamics to be altered. Using a fixed time scale, we can numerically model a particular glacier with a geometrically similar one that is 10 times the original dimensions in all directions; but this is only possible if gravity is also 10 times stronger. Conversely, if we are studying ice in a centrifuge (or perhaps on a substantially larger planet), then a 1000‐fold increase in gravity means we can study the same glacier at one tenth of its original size and in one tenth the time. Of course, a tenfold or 1000‐fold increase in gravity would make accelerations of ice nonnegligible, and these dimensionless parameters are perfectly capable of handling that situation. By preserving D*u*/D*t* in equation (15), we have not made any assumptions about the magnitude of gravity or the significance of acceleration of flowing ice within the equations of motion.

Note, however, that Glen's flow law may or may not apply to accelerating ice, and this illustrates the distinct advantage of using a stretching symmetry analysis rather than Buckingham Pi. With stretching symmetries, we identify the role of each dimensionless group according to its physical origin. We can see that rescaling gravity in a numerical model is plausible and useful, but whether it is physically appropriate must be decided on the basis of Glen's flow law. Only an understanding of the underlying physics allows us to make this distinction.

Our derivation of the dimensionless parameters (103), (104), (105), (106), (107), (108), (109), (110) has been exact. There have been no assumptions except for our choice of Glen's flow law as the constitutive equation. We particularly emphasize that the derivations have *not* assumed any of the commonly adopted simplifying assumptions, such as plane strain, steady state conditions, perfect plasticity, or shallow‐ice approximations.

### 6.2 Characteristic Values

Equation (108) is a restatement of response time scaling. Rearranging and selecting *i* = *z* gives a more familiar form

(111) $$t={\Pi_{6}}^{\prime }\frac{u_{z}}{z}$$

If we select the characteristic vertical velocity as $\overset{\text{˙}}{b}$, the characteristic depth as the thickness *h*, and the characteristic time as the *e*‐folding volume response time *τ*, then

(112) $$\tau ={\Pi_{6}}^{\prime }\frac{h}{\dot{b}}$$

as derived by *Jóhannesson et al*. [1989], *Bahr et al*. [1998], *Harrison et al*. [2001], and *Raper and Braithwaite* [2009] among others.

Like all dimensional relationships, the values assigned to each variable in equation (111) are characteristic quantities, and there are no single correct choices. *Jóhannesson et al*. [1989] assigned *u_z_* as the scale of ablation at the terminus and *z* as the average ice thickness, a reasonable choice for estimates of the *e*‐folding volume response time. However, the nature of the application dictates the selection, just as it does when choosing a characteristic length for Reynolds number in Newtonian fluid flow. Selecting the appropriate magnitudes for terms in dimensionless parameters remains very much a matter of judgment. For example, in turbulent pipe flow, the diameter of the pipe is the relevant length scale, while for turbulent flow around an obstacle, the length of the obstacle is the relevant scale.

Similarly, the characteristic time in (111) could be the *e*‐folding volume response time (as commonly assumed) or it could be the interval between large snow storms. The former is useful when predicting a glacier's response to climate, and the latter is useful when discussing transient changes in thickness from snowfall events. Likewise, the characteristic thickness could be the average glacier thickness, the average thickness at the equilibrium line, the thickness at a particular point, the average thickness of a snowfall event, the thickness of a large bump on the bed, or many other possible measures of vertical scale. The characteristic vertical velocity can likewise be the average balance rate above the equilibrium line, the balance rate at the terminus, or another choice judged to be appropriate for the particular problem at hand.

In general, all of the variables in the dimensionless relations are characteristics and should be assigned values relevant to the particular problem. Even gravity is a characteristic value. We can assign 9.8 m/s^2^, or we can assign a value appropriate to an experiment with ice in a centrifuge (assuming Glen's flow law applies to an ice sample in a centrifuge). Similarly, the velocity in (110) can be the average velocity of the entire glacier, the average velocity at the equilibrium line, the average velocity around a nunatak, the average basal sliding velocity, or some other velocity altogether. The length scale could be the length of the entire glacier, the length of a nunatak, or innumerable other choices that can only be determined after a particular problem is identified.

If the intuitive choice of characteristic values seems in any way arbitrary, it is worth noting that characteristic values frequently can be incorporated as a part of the stretching analysis. This has already been illustrated with the continuity equation. In equation (12), the thickness does not appear explicitly, but in its integrated form (13), the thickness appears naturally as a part of the integrands. Similarly, if the basal shear stress is the desired characteristic value, then a vertical integration of the equations of motion from the basal boundary to the surface will introduce the basal shear; a stretching symmetry of this vertically integrated equation will produce a set of stretching constraints that contain the basal shear explicitly. Likewise, the basal sliding velocity could be introduced through an integration of momentum conservation. Carefully selected integrals can introduce characteristic averages values, total values, and/or specific values via the integrand.

The choice of characteristic values will be relevant when deriving the volume‐area scaling exponent. We will clearly be interested in characteristic values that give some reasonable measure of the average glacier thickness, the average length, and the average width. Together, these will define a volume applicable to the entire glacier.

Similarly, we will want a characteristic area that reflects the total surface area of the glacier. This characteristic could be the actual surface area, the area of a horizontal map projection, the accumulation area, the ablation area, or many other possibilities. The actual surface area is an obvious choice and the one used in the following derivations. However, other choices lead to straightforward modifications. For example, if we instead selected the accumulation area, then the total area *S* is related to the accumulation area *S*_acc_ by *S*_acc_ = AAR × *S* where AAR is the ratio of the accumulation area to the total area. By substitution into equation (1), the multiplicative scaling parameter *c* will change by a factor of 1/AAR^*γ*^. Similarly, the horizontally projected map plane area would modify *c* with a projection angle that changes from glacier to glacier. The role of the glacier slope is discussed in the following derivations, and its inclusion as a variable projection angle would follow in an obvious manner.

### 6.3 Choosing Dimensionless Parameters

In fluid dynamics it is common to choose a dimensionless parameter Π_i_ that is most relevant to a particular problem. If estimating turbulence, then Reynolds' number is used. If measuring drag characteristics on the hull of a ship, then Froude's number is used. However, it is not uncommon to require both, or even all, possible parameters. For example, turbulent flow around a hull will require both Froude's number and Reynolds' number. If there is any doubt, the important variables should be defined and then a Buckingham Pi analysis will indicate the necessary set of relevant parameters.

Similarly, we can choose a subset of dimensionless parameters that are most relevant to a particular glaciological problem, but ideally, a Buckingham Pi analysis should be used to avoid uncertainties. For example, time may or may not be a relevant variable, depending on whether or not the glacier is in steady state. If the glacier is not in steady state, then an application of the Buckingham Pi Theorem confirms that we must include response time scaling (108).

For volume‐area scaling applications, this will be particularly important. Volume‐area scaling does not explicitly include a variable for time, but volume‐area scaling can still be applied to transient and nonsteady state conditions—the temporal component is included via the separate but equally relevant response time scaling. These two scaling relationships cannot be artificially separated. Claiming that volume‐area scaling is somehow separate from response time scaling is exactly analogous to claiming that “the stress equilibrium equations do not explicitly include time, and therefore, stresses cannot change with time.” That is obviously nonsensical because the separate but equally important continuity equation includes the temporal component. In other words, the nonsteady state temporal behavior of volume‐area scaling is encapsulated in the separate response time scaling, as confirmed by an application of the Buckingham Pi Theorem.

## 7 Volume‐Area Scaling From Dimensionless Parameters

Many different glaciological problems are amenable to a dimensional analysis, but volume estimation is the most common. Therefore, the following derives specific volume‐area scaling equations. The approach used here could be generalized to derive a wide variety of other scaling relationships between other continuum variables.

Importantly, *Bahr* [1997a] has shown that any scaling relationship involving two fundamental glaciological continuum variables (see section 4) can be derived directly from volume‐area scaling (e.g., see section 8.3). In other words, all scaling relationships are closely related to one another, and a derivation of one implies many of the others. This theory is put to practical use in *Bahr et al*. [2014]. While volume‐area scaling may appear to be a narrow and specific application of the dimensional analysis, its derivation has wide reaching implications.

The derivations in sections 7.1 and 7.2 start with combinations of relevant dimensionless groups. These combinations dictate the form of the multiplicative scaling parameter *c* in equation (1). When applied to large collections of glaciers, *c* will have many different values because each glacier has a different set of dimensionless parameters. In other words, the dimensionless parameters are not invariant so neither is *c*. However, the 1 order of magnitude variation in *c* [e.g., *Bahr*, 1997a] is small compared to the range of possible values of glacier area *S* (4 orders of magnitude) and glacier volumes *V* (5 orders of magnitude). Therefore, despite the scatter in *c*, volume and area have a readily discernable power law trend, as seen in Figure 1. This differs fundamentally from a shallow‐ice approximation that assumes the relevant dimensionless parameter must remain unchanged for all glaciers. The assumptions built into the shallow‐ice approximations are not necessary in the following derivation.

### 7.1 Volume‐Area Scaling for Glaciers

The derivation that follows is similar to but more general than *Bahr et al*. [1997]. A volume‐area scaling relationship can be derived by combining the dimensionless parameters given above and by selecting appropriate characteristic values. From a combination of (26) and (28),

(113) $$\frac{\Pi_{4}}{\Pi_{2}}=\frac{u_{x}z}{u_{z}x}$$

Note that (113) is “directionally consistent” and independent of the axes orientation (see section 4.2). The dimensionless parameter Π_9_ in (33) can be combined with (113) to eliminate *u_x_*.

(114) $$\frac{\Pi_{4}\Pi_{9}}{\Pi_{2}}=\frac{A\rho^{n}{g_{x}}^{n}z^{n+2}}{u_{z}x}$$

Again, this expression is valid for any orientation of the axes. Equation (114) does not need nor should contain any reference to a particular angle or slope. This helps reduce the number of closure conditions used below because a “slope” closure is unnecessary.

Choose the length of the glacier *l* as the characteristic length, $\overset{\text{˙}}{b}$ at the terminus as the characteristic vertical velocity, the average width *w* as the characteristic width, and the average thickness *h* as the characteristic thickness. The exact choice of characteristic values is almost irrelevant because they cancel when solving for the area and volume; however, these choices are motivated by their relationship to known closure conditions. The closure conditions are imposed scaling relationships motivated by observations. Similar to a boundary condition in continuum mechanics, at least one closure condition is necessary in the following derivation.

For example, data show that average width scales with average length.

(115) $$w=c_{q}l^{q}$$

where *q* ≈ 0.6 = 3/5 for glaciers and *q* = 1 for ice caps [*Bahr*, 1997b]. The value of this exponent *q* reflects the relationship between the geometry of the glacier itself and the underlying topography. Because an ice body would creep outward in a radially symmetric pattern from a center of mass input if there were no underlying topography to impose an asymmetry on the flow, *q* = 1 is a logical value for an ice cap, where the subglacial topography no longer has a significant influence on the overall shape of the body. A similar scaling is applied to the relationship between mass balance and length:

(116) $$\dot{b}=c_{m}l^{m}$$

where *m* ≈ 2 for glaciers and *m* ≈ 0 for ice caps [*Bahr et al*., 1997; *Bahr and Dyurgerov*, 1999]. This is an expression of how the length (or size) of a glacier controls the magnitude of the balance. Because larger glaciers cover a wider altitude range, their termini reach lower into the ablation area and thus encounter an increasingly negative characteristic balance at the terminus.

Substituting the characteristic values and balance closure condition into (114) gives

(117) $$h={\left(\frac{\Pi_{4}\Pi_{9}c_{m}}{\Pi_{2}A\rho^{n}{g_{x}}^{n}}\right)}^{\frac{1}{n+2}}l^{\frac{m+1}{n+2}}$$

Volume *V* can be expressed as a product of the characteristic length, width, and thickness. Surface area *S* is a product of characteristic width and length. Therefore, from width scaling (equation (9)) and from (117)

(118) $$\begin{array}{c} V=l\;w\;h\\ =l\left(c_{q}l^{q}\right)\left({\left(\frac{\Pi_{4}\Pi_{9}c_{m}}{\Pi_{2}A\rho^{n}{g_{x}}^{n}}\right)}^{\frac{1}{n+2}}l^{\frac{m+1}{n+2}}\right)\\ =c_{q}{\left(\frac{\Pi_{4}\Pi_{9}c_{m}}{\Pi_{2}A\rho^{n}{g_{x}}^{n}}\right)}^{\frac{1}{n+2}}l^{1+q+\frac{m+1}{n+2}} \end{array}$$

Similarly,

(119) $$\begin{array}{c} S=l\;w\\ =l\left(c_{q}l^{q}\right)\\ =c_{q}l^{1+q} \end{array}$$

By combining (118) and (119), we arrive at the volume‐area scaling relationship, *V* = *cS* ^*γ*^:

(120) $$\begin{array}{c} V=c_{q}{\left(\frac{\Pi_{4}\Pi_{9}c_{m}}{\Pi_{2}A\rho^{n}{g_{x}}^{n}}\right)}^{\frac{1}{n+2}}{\left(\frac{S}{c_{q}}\right)}^{1+\frac{m+1}{\left(n+2\right)\left(q+1\right)}}\\ ={c_{q}}^{\frac{-\left(m+1\right)}{\left(n+2\right)\left(q+1\right)}}{\left(\frac{\Pi_{4}\Pi_{9}c_{m}}{\Pi_{2}A\rho^{n}{g_{x}}^{n}}\right)}^{\frac{1}{n+2}}S^{1+\frac{m+1}{\left(n+2\right)\left(q+1\right)}} \end{array}$$

where the scaling parameter *c* is completely defined as

(121) $$c={c_{q}}^{\frac{-\left(m+1\right)}{\left(n+2\right)\left(q+1\right)}}{\left(\frac{\Pi_{4}\Pi_{9}c_{m}}{\Pi_{2}A\rho^{n}{g_{x}}^{n}}\right)}^{\frac{1}{n+2}}$$

and the scaling exponent *γ* is

(122) $$\gamma =1+\frac{m+1}{\left(n+2\right)\left(q+1\right)}$$

This is identical to the exponent derived in *Bahr et al*. [1997] except that, in this derivation, no closure condition for slope is necessary. (To compare with *Bahr et al*. [1997], set *r* = 0 and *f* = 0 in their analysis, as they do.) The directional compatibility analysis in section 4.2 makes it clear that angles and slopes are not necessary.

In fact, only one closure condition is strictly necessary for *γ*, and we can also remove either the mass balance scaling exponent *m* or the width scaling exponent *q*. From our earlier analysis with equation (11), *γ* = (2*q* + 1)/(*q* + 1). Therefore, substituting into (122) shows

(123) $$m=q\left(n+2\right)-1$$

Thus, for *n* = 3

(124) q=3/5⇔m=2

The values of *q* and *m* are not independent, and therefore, these two closure conditions are equivalent. It is encouraging that independent data for width and mass balance scaling support both values [*Bahr*, 1997b; *Bahr et al*., 1997; *Bahr and Dyurgerov*, 1999].

Also, note that for most glaciers (not ice caps and not many tropical glaciers) the accumulation area ratio or AAR (ratio of accumulation area to total glacier area) can be reasonably approximated by

(125) $$AAR={\left(\frac{1}{m+1}\right)}^{\frac{1}{m}}$$

(This is derived in *Bahr et al*. [1997] by using the shape of the balance closure in (116) to identify the position of the equilibrium line.) For a glacier in equilibrium, data show that the AAR ≈ 0.57 [*Bahr et al*., 2009; *Dyurgerov et al*., 2009; *Mernild et al*., 2013]. Evaluating (125) at *m =* 2 gives AAR = 0.57; thus,

(126) q=3/5⇔m=2⇔AAR=0.57

Choosing any one of these implies the others, and data independently support all three.

In summary, we can write three different expressions for the glacier volume‐area scaling exponent *γ*:

(127) $$\begin{array}{c} \gamma =1+\frac{q}{q+1}\\ =1+\frac{m+1}{m+n+3}\\ =1+\frac{{AAR}^{-1}\left(m\right)+1}{{AAR}^{-1}\left(m\right)+n+3} \end{array}$$

where AAR^− 1^(*m*) is the functional inverse of (125). Independent data available for all three expressions [*Bahr*, 1997b; *Bahr et al*., 1997; *Bahr and Dyurgerov*, 1999; *Bahr et al*., 2009; *Dyurgerov et al*., 2009; *Mernild et al*., 2013] give the same result of *γ* = 1.375. For the given values of *q, m,* and AAR, this value of *γ* is fixed and is not a variable free for tuning.

Note that the scaling parameter *c* in equation (121) contains three dimensionless parameters Π_2_, Π_4_, and Π_9_. As in other applications, these dimensionless parameters are not fixed constants and have a distribution of possible values. It is theoretically possible but highly improbable that the unknown values for *c*_*q*_and *c*_*m*_ will always cancel the variations in Π_2_, Π_4_, and Π_9_. This is highly unlikely because *c*_*q*_ and *c*_*m*_ are imposed as external closure conditions that are separate from the dimensionless parameters which are derived from the continuity equation, constitutive equation, and equations of motion. Therefore, the scaling parameter *c* will have a distribution of possible values and, unlike *γ*, should *not* be treated as a constant (cf. the appendix of *Bahr* [1997a]).

Importantly, basal sliding and other boundary conditions cannot change the scaling exponent as discussed above, but these boundary conditions could have a very important influence on the random distribution of *c*. For example, if basal sliding dominates most small glaciers, this could introduce a characteristic (sliding) velocity to Π_9_. In turn, the values of Π_9_ will change *c*.

### 7.2 Volume‐Area Scaling for Ice Caps

As discussed above, the closure conditions for volume‐area scaling for an ice cap and ice sheet are not the same as those used for glaciers. For a glacier, the topography is controlled by a valley's shape; but for an ice cap, the bedrock topography is submerged beneath the ice cover and has little or no influence on the ice geometry. The derivation of the AAR in equation (125) assumes that the glacier has a predominately asymmetric, linear shape, but for an ice cap, the shape is radially symmetric.

For the moment, assume that the fundamental difference between an ice cap and a glacier is that the former substantially subsumes its bedrock topography while a glacier does not. Specifically, the vertical relief of the ice cap (i.e., the ice thickness) is large compared to the vertical relief of the underlying topography, while the ice thickness of the glacier is small compared to the relief of the surrounding bedrock topography. Under these conditions, the surface slope *θ* of an ice cap is defined primarily by changes in ice thickness d*h/*d*x*. It follows that, with some assumptions about the mass balance profile (either constant across the glacier or a step function from the accumulation to ablation area), a well‐known parabolic surface profile can be derived [e.g., *Nye*, 1951; *Cuffey and Paterson*, 2010]. The scaling exponent follows trivially. In particular, if *h* ∝ *l* ^1/2^ (parabolic surface profile) and *w* ∝ *l* (radial symmetry), then

(128) $$\begin{array}{c} V=l\;w\;h\\ \propto l\cdot l\cdot l^{1/2}\\ =l^{5/2}\\ \propto S^{5/4} \end{array}$$

The scaling exponent is *γ* = 5/4 = 1.25, the exponent widely accepted for ice caps [e.g., *Cuffey and Paterson*, 2010; *Radić and Hock*, 2010].

More generally, the derivation of *Bahr et al*. [1997] uses the height/length parameter

(129) θ∝z/x

without explicitly requiring a parabolic surface profile and without requiring a thickness that is large compared to underlying topography. This relationship (129) is required by a directional analysis (see section 4.2). Equation (129) is also an obvious dimensional consequence of sin*θ =* d*h/*d*x*, but that relationship is not required in the following derivation*.* Combining (129) with an analysis similar to the one above for glaciers (see equations (113), (114), (115), (116), (117), (118), (119), (120)).

(130) $$\begin{array}{c} \frac{\Pi_{2}\Pi_{9}}{\Pi_{4}{\Pi_{6}}^{n}}=\frac{A\rho^{n}{g_{x}}^{n}z^{n+2}}{u_{z}x}\\ =\frac{A\rho^{n}{g_{z}}^{n}\theta^{n}z^{n+2}}{u_{z}x} \end{array}$$

as required by directional consistency (see equation (49) in section 4.2). Substituting (129) and substituting characteristic values,

(131) $$h={\left(\frac{\Pi_{2}\Pi_{9}c_{m}}{\Pi_{4}{\Pi_{6}}^{n}A\rho^{n}{g_{z}}^{n}}\right)}^{\frac{1}{2\left(n+1\right)}}l^{\frac{1}{2}+\frac{m}{2\left(n+1\right)}}$$

and

(132) $$\begin{array}{c} V=l\;w\;h\\ =c_{q}{\left(\frac{\Pi_{2}\Pi_{9}c_{m}}{\Pi_{4}{\Pi_{6}}^{n}A\rho^{n}{g_{z}}^{n}}\right)}^{\frac{1}{2\left(n+1\right)}}l^{1+q+\frac{1}{2}+\frac{m}{2\left(n+1\right)}}\\ =c_{q}{\left(\frac{\Pi_{2}\Pi_{9}c_{m}}{\Pi_{4}{\Pi_{6}}^{n}A\rho^{n}{g_{z}}^{n}}\right)}^{\frac{1}{2\left(n+1\right)}}{\left(\frac{S}{c_{q}}\right)}^{1+\frac{m+n+1}{2\left(n+1\right)\left(q+1\right)}}\\ ={c_{q}}^{\frac{-\left(m+n+1\right)}{2\left(n+1\right)\left(q+1\right)}}{\left(\frac{\Pi_{2}\Pi_{9}c_{m}}{\Pi_{4}{\Pi_{6}}^{n}A\rho^{n}{g_{z}}^{n}}\right)}^{\frac{1}{2\left(n+1\right)}}S^{1+\frac{m+n+1}{2\left(n+1\right)\left(q+1\right)}} \end{array}$$

For a radially symmetric ice cap (as well as elliptical ice cap), *q* = 1. Substituting this into the scaling exponent in (132) gives

(133) $$\gamma =1.25+\frac{m}{4\left(n+1\right)}$$

When *m* = 0, making $\overset{\text{˙}}{b}$ independent of length, as commonly assumed for an ice sheet [e.g., *Cuffey and Paterson*, 2010; *Meehl et al*., 2007], *γ* = 1.25 for any value of *n*. Furthermore, for *m* ≪ 1, the scaling exponent will remain near 1.25 because the denominator in (133) is large. Also, note that as *n* → ∞ (a perfectly plastic ice sheet), the scaling exponent becomes 1.25 for any value of the closure condition *m*. However, perfect plasticity is not necessary to derive the ice cap (or glacier) scaling exponent.

Note that the structure of the scaling parameter *c* for ice caps is similar, but not identical, to the parameter derived for glaciers:

(134) $$c={c_{q}}^{\frac{-\left(m+n+1\right)}{2\left(n+1\right)\left(q+1\right)}}{\left(\frac{\Pi_{2}\Pi_{9}c_{m}}{\Pi_{4}{\Pi_{6}}^{n}A\rho^{n}{g_{z}}^{n}}\right)}^{\frac{1}{2\left(n+1\right)}}$$

For a radially symmetric ice cap, width and length are identical, and *c_q_* = 1. As with glaciers, the dimensionless parameters Π_2_, Π_4_, Π_6_, and Π_9_ have distributions of values. As a result, *c* will also have a distribution of values and is not a constant.

### 7.3 Alternative Closure Conditions

At least one closure condition is necessary in the preceding derivations of volume‐area scaling. Each of the three closures used above lead to identical results. Each one imposes a scaling relationship that summarizes a geometric or a mass balance condition. In continuum mechanics, analogous boundary conditions for velocity and/or stress are equally important. We could reasonably ask, therefore, if a different closure condition related to velocity or stress (or any other parameter) could change the scaling exponent.

For example, a hypothetical sliding closure condition could relate characteristic basal velocity to another characteristic quantity such as surface area, volume, or thickness. We do not currently have the observations available to do this. However, we have shown that three other known closure conditions lead to the same scaling exponent of 1.375 (which is also consistent with observations of volume and area data). Only one closure conditions is necessary, as shown in the derivation. Therefore, if the proposed basal sliding closure generated a different exponent *γ*, then we would be forced to explain why data for all of the width, mass balance, and AAR closures are wrong, and then also explain why the available volume‐area observations are wrong. It is very unlikely that four sets of independent observations are all wrong. Occam's razor suggests that the more likely scenario is that any proposed sliding closure will be consistent with the existing width, mass balance, and AAR closure conditions as well as with the volume‐area observations. For the same reason, any other proposed closure condition is unlikely to change the scaling exponent.

## 8 Discussion

The various applications of volume‐area scaling in the glaciological literature have suffered from a number of misapplications and misunderstandings about the generality of the scaling theory. Here we address a number of these problems specifically and clarify points of application that will improve the value of future analyses using these methods. The subsequent sections each address some particular characteristic of volume‐area scaling that has been misinterpreted at some point in the literature (e.g., Table 1).

### 8.1 Volume‐Area Scaling Is Derived Without Assumptions of Plane Strain, Steady State, Perfect Plasticity, or Shallow‐Ice Approximations

Although various publications state otherwise [e.g., *Adhikari and Marshall*, 2012, p. 3; *Farinotti et al*., 2009, p. 428; *Radić et al*., 2007; *Meehl et al*., 2007, p. 815], no assumptions about plane strain, shallow ice, perfect plasticity, or steady state conditions are necessary in the derivation of the volume‐area scaling exponent. The dimensionless parameters were derived from the full set of continuum equations using two different and independent techniques (dimensional analysis and stretching symmetries). The scaling exponents of *γ =* 1.375 (glaciers) and *γ =* 1.25 (ice caps) are completely determined by the physics, up to the choice of a single closure condition. This closure condition is generated from data, but three possible closure choices for glaciers (the width‐length scaling exponent, the mass balance scaling exponent, and the equilibrium value of the AAR) give the same result. Each closure can be derived from the others, and the three choices are internally consistent both in theory and data (see (126)).

### 8.2 The Volume‐Area Scaling Exponent *γ* Is a Constant and the Parameter c Is Not a Constant

For most applications, the scaling exponent *γ* should be treated as a constant, and the scaling parameter *c* should *not* be treated as a constant. The parameter *c* can have a distribution of possible values, but the exponent *γ* has a single value that is fixed by the underlying physics plus minimal empirical quantification of the closure conditions; any hypothesized changes in *γ* would require changes in the closure conditions, which are in turn fairly tightly constrained by observed glacier behavior. Adjustments to the recommended values of *γ* = 1.375 and 1.25 are possible but improbable given that three separately measured physical quantities (width closure, mass balance closure, and AAR closure) lead to the same result. In spite of this, the scaling exponent *γ* is widely regarded as an adjustable parameter [*Grinsted*, 2013; *Adhikari and Marshall*, 2012; *Radić et al*., 2007; *Bliss et al*., 2013; *Hagg et al*., 2013] although none of those analyses have explained why variations in *γ* do not lead to inconsistencies between the various closure conditions or (even worse) inconsistencies in the underlying continuum equations. Any application (e.g., sea level rise) that uses an alternative or variable value for *γ* should include a rigorous justification using closure data, the underlying physics, or convincing data.

The range of possible variation in *γ* can be constrained by straightforward and robust conditions. If no closure or boundary conditions were imposed, then a simple dimensional analysis would relate *V* ∝ *l* ^3^ to *S* ∝ *l* ^2^ and thus *γ* = 3/2. This value would apply to the classic continuum‐mechanical “control volume” [e.g., *Welty et al*., 1984], when far from the influence of any glacier boundaries. This simple assessment establishes *γ* = 3/2 as an upper bound.

More generally, the width scaling exponent *q* for glaciers cannot be larger than 1 (a fully two‐dimensional glacier surface) and cannot be smaller than 0 (a one‐dimensional glacier surface). If 0 ≤ *q* ≤ 1, then from equation (127), this implies 1 ≤ *γ* ≤ 3/2.

Similarly, 0 ≤ AAR ≤ 1 by definition. Therefore, from (125), − 1 ≤ *m* ≤ ∞. Combining with (127) gives 1 ≤ *γ* ≤ 2. This is less restrictive than the constraint imposed by *q*. However, from (123) the maximum for the mass balance scaling exponent is *m* = 4 when *q* = 1, and therefore 1 ≤ *γ* ≤ 3/2 as above.

Physical reasoning suggests that *m* is most likely greater than 0. Values of *m* < 0 would make the characteristic balance rate diverge toward infinity for very small glaciers because the characteristic balance becomes inversely proportional to the characteristic length (per equation (116)). This is highly unlikely. Therefore, 0 ≤ *m* ≤ 4, and for glaciers it follows that

(135) 7/6≤γ≤3/2

or 1.1667 ≤ *γ* ≤ 1.5. These simple considerations place bounds on reasonable choices of *γ*.

For ice caps, reasonable bounds are more obvious. If *m* = 0 is the smallest possible value, then from equation (133), the smallest possible volume‐area scaling exponent is 5/4. As with glaciers, the largest possible ice cap scaling exponent is 3/2.

As an example, we might propose that *γ* = 1.46 for glaciers [cf. *Adhikari and Marshall*, 2012] which is within the range of possible values. In that case, from (127), we derive *q* = 0.85, *m* = 2.4, and AAR = 0.6. These closure conditions are not particularly good matches with observations for *q*, *m*, and AAR, but more importantly, *q* = 0.85 and *m* = 2.4 are inconsistent with equation (123). Some explanation of these inconsistencies would need to be proposed.

Similarly, we might propose *γ =* 1.22 for ice caps [cf. *Meier and Bahr*, 1996; *Grinsted*, 2013]. This falls outside the range of likely values and requires negative values for the mass balance scaling exponent. From equation (133), *γ =* 1.22 requires *m* = −0.48.

*Radić et al*. [2007] use the shallow‐ice approximation in a one‐dimensional flow line model to find *γ =* 1.56. This is outside the range of likely values, and the use of a shallow‐ice approximation may have limited the applicability of their results. Numerical models that employ simplified continuum equations can lead to different scaling exponents.

*Grinsted* [2013] has compiled an excellent summary of volume‐area scaling exponents reported in the literature. Many fall within reasonable bounds, but a few of the estimates fall outside the range given above. For those estimates that are significantly different from the theory, none of the references offer an explanation that is not in conflict with the theory—for example, nonequilibrium conditions cannot explain the difference because the theory does not assume steady state conditions and the scaling exponent is not a function of time, as discussed below.

### 8.3 The Scaling Exponent *γ* Is Not a Function of Time

Some studies have suggested that the volume‐area scaling exponent varies as a function of time, depending, for example, on the extent to which a glacier is out of equilibrium [e.g., *Adhikari and Marshall*, 2012; *Radić et al*., 2007]. This is not the case. If volume‐area scaling is a function of time, then a scaling analysis shows that the characteristic *e*‐folding response time must also be a function of time. By definition, however, the characteristic response time is a constant *τ* in an exponential decay. This leads to a contradiction that can only be resolved if *γ* is a constant.

In particular, select *z*, *u*_*x*_, and *σ*_*xz*_ as a set of basis vectors that span each of the fundamental dimensions in the dimensional matrix (Table 2). Following the methods of *Bahr* [1997a], each of these three quantities can be scaled as *S* ^*f*(*γ*,*n*,*q*)^, where *f* is some rational polynomial function of the volume‐area scaling exponent, the flow law exponent, and one of the closure condition exponents (in this case *q*). Using the Buckingham Pi Theorem, this implies that all of the other continuum mechanical variables can also be written as *S* ^*f*(*γ*,*n*,*q*)^.

Select any two arbitrary continuum variables *φ* and *ψ*. From the above,

(136) $$\varphi =c_{\varphi }S^{f\left(\gamma ,n,q\right)}$$

and

(137) $$\psi =c_{\psi }S^{g\left(\gamma ,n,q\right)}$$

for some scaling parameters *c*_*φ*_ and *c*_*ψ*_ and rational polynomial functions *f* and *g*. These can be combined as

(138) $$\psi =c_{\psi }{\left(\frac{\varphi }{c_{\varphi }}\right)}^{\frac{g\left(\gamma ,n,q\right)}{f\left(\gamma ,n,q\right)}}$$

In other words, all possible scaling relationships between any two continuum variables can be expressed as (138).

From (138), if the volume‐area scaling exponent *γ* is a function of time, then all other possible scaling exponents for any scaling relationships between two continuum variables must also be functions of time (except in the few instances that *f* and *g* are identical up to a multiplicative constant). This already violates the spirit of the Buckingham Pi Theorem (and the stretching symmetry analysis) that always derives *constant* scaling exponents. See, for example, any of the Π_*i*_ in equations (25), (26), (27), (28), (29), (30), (31), (32), (33), (34), (35), (36), (37), (38), (39).

We can derive an explicit contradiction by considering the *e*‐folding volume response time *τ* that is defined as the exponential rate *constant* in an exponential decay.

(139) $$V\left(t\right)=V_{0}+\Delta V\left(1-e^{-t/\tau }\right)$$

where *V*_0_ is the initial volume and Δ*V* is a volume perturbation. Note that the time dependence is stated explicitly and *τ* is not a function of time. Although equation (139) is the most commonly used functional form of an *e*‐folding decay [e.g., *Jóhannesson et al*., 1989], the exact formulation is not critical and could be generalized in many ways. By replacing *e* with 2, the equation could instead be defined as a half‐life (the time to decay to half the original volume), or the equation could be replaced by a decreasing power law, inverse logarithm, or other decay function. The key point is that in any proposed decay equation, the characteristic response time *τ* must be a constant with respect to time. (Note that other time scales could be defined that do vary with *t*. However, these scales would not be the direct result of a characteristic scaling transformation in the sense defined by stretching symmetries where (by definition) $\bar{t}=t/\tau$ for constant *τ*. See equation (50)).

From (112) and following a derivation similar to *Bahr et al*. [1998] and *Pfeffer et al*. [1998],

(140) $$\tau \propto \frac{\dot{b}}{h}\propto \frac{S^{\gamma -1}}{l^{m}}\propto \frac{S^{\gamma -1}}{S^{\frac{m}{q+1}}}\propto S^{\gamma -1}S^{\frac{-m}{\left(\frac{m+1}{n+2}\right)+1}}\propto S^{\left(\gamma -1\right)-\left(\frac{m\left(n+2\right)}{m+n+3}\right)}$$

As expected, this has the form of (136). It is immediately apparent that if *γ* is a function of time, then *τ* must also be a function of time. However, we already know that *τ* is defined as a constant in (139).

This contradiction can be resolved if *λ* − 1 = (*m*(*n* + 2))/(*m* + *n* + 3). In this case, the exponent in (140) disappears and *τ* is no longer dependent on *γ*. However, for any reasonable values of *γ*, *m* and *n*, this is not possible (see previous section), nor is it intuitively likely – we expect large glaciers to have different response times than small glaciers. Therefore, the only remaining conclusion is that *γ* must not be a function of time.

More generally, it follows from (138) that no scaling exponents for any scaling relationship can be a function of time. This key conclusion suggests that additional research should focus on understanding the potential time dependence of scaling parameters such as *c*. If observations or models suggest any time dependence in volume‐area scaling or other scaling relationships, changes in these scaling parameters (such as *c*) are the most likely explanation.

### 8.4 Volume‐Area Scaling Applies to Nonequilibrium Conditions With the Inclusion of Response Time Scaling

Although volume‐area scaling is occasionally claimed to be an equilibrium‐only theory [e.g., *Adhikari and Marshall*, 2012; *Farinotti et al*., 2009, p. 428], volume‐area scaling has been derived using the fully time‐dependent equations of motion. Even though *γ* cannot change with time (see section 8.3), each of the other terms *V*, *A*, and *c* can vary with time. Although often overlooked, much of the unknown transient behavior can be incorporated into the scaling parameter *c*. As shown in equations (121) and (134), the scaling parameter has an explicit dependence on dimensionless parameters that are known to vary with time. Closure condition parameters *c_m_* and *c_q_* in these same equations might also vary with time.

Furthermore, while the fundamental volume‐area scaling equation (1) does not show time dependence explicitly, the relevant time dependence is summarized within response time scaling, such as equations (112) and (140). Volume‐area scaling and response time scaling coexist, act in concert, and are derived simultaneously using the Buckingham Pi Theorem (and/or stretching symmetries). The effects of these two scaling relationships cannot be artificially separated as noted in section 6, and the interplay between these two scaling relationships is illustrated in a sea level rise application by *Marzeion et al*. [2012] as well as in the theoretical work of *Lüthi* [2009] and *Raper and Braithwaite* [2009].

The response time work by *Harrison et al*. [2001, 2003] and *Harrison* [2013] also suggests an effective means of combining response time scaling with volume‐area scaling. The thickness parameter *H* in *Harrison et al*. [2003, equation (6)] and *Harrison* [2013, equation (6)] can be converted to the notation of this review and rescaled as *H* ≡ *γh*. In that case, their thickness parameter matches the derivative of the volume‐area scaling relationship, d*V*/d*S* = *γh* (see equation (1)). Although their results are intended for individual glaciers rather than ensembles of glaciers, their derivations and results suggest a close interaction between glacier response time and volume‐area relationships. In particular, their time scales for individual glaciers are broadly compatible with the time scales for ensembles of glaciers as presented in this review. Their work would suggest a rescaling of the thickness and volume time scales of *Jóhannesson et al*. [1989], along the lines illustrated by *Raper and Braithwaite* [2009, equation (26)].

Interactions of volume‐area scaling and response time scaling have also been explored in the numerical experiments of *Pfeffer et al*. [1998]. As in the theoretical works by *Harrison* [2013], *Lüthi* [2009], and *Raper and Braithwaite* [2009], the numerical modeling experiments confirm that response time scaling and volume‐area scaling are related and inseparable.

### 8.5 Volume‐Area Scaling Applies to Populations of Glaciers, and Not to Individual Glaciers

Volume‐area scaling can lead to very large errors when applied to a single glacier for which the scaling parameter *c* has not been established [*Agrawal and Tayal*, 2013; *Barrand and Sharp*, 2010; *Meier et al*., 2007; *Schneeberger et al*., 2003; *Farinotti and Huss*, 2013]. For example, for any particular glacier we might reasonably select the well‐defined worldwide mean of the scaling parameter *c* = 0.034 km^3 − 2*γ*^ (from the roughly normal distribution in *Bahr* [1997a]). Suppose, however, that the correct value for this particular glacier is different from the mean by an amount Δ*c*. From equation (1), this creates a volume error Δ*V* given by

(141) $$V+\Delta V=\left(c+\Delta c\right)S^{\gamma }$$

Subtracting *V* = *cS* ^*γ*^ from each side gives

(142) $$\Delta V=\;\Delta c\;S^{\gamma }$$

Dividing by *V* then gives the fractional error

(143) $$\frac{\Delta V}{V}=\;\frac{\Delta c}{c}$$

The fractional error in a glacier's estimated volume is directly proportional to the fractional error in *c*. Thus, a 10% error in the scaling parameter *c* will lead to a 10% error in the volume, independent of the glacier's size. When using the mean value of *c* for a single randomly selected glacier, a one standard deviation error in *c* is certainly possible and would produce a 34% error in volume. These errors are distinct from the additional influence of surface area measurement errors. For this reason, volume scaled from the area of an individual glacier can be accurately treated as an order of magnitude estimate, but any additional precision must be carefully evaluated in the context of potentially large errors. We do not recommend applying volume‐area scaling to individual glaciers without careful justification and assessment of the error. This limitation was stated in several early papers of volume‐area scaling [*Chen and Ohmura*, 1990], but is nonetheless widely unrecognized.

Scaling can give accurate estimates of the aggregate volume of *ensembles* of glaciers. The random errors in *c* and *S* create a distribution of volumes for some distribution of areas, but the mean and variance will be well‐defined by the means and variances of both *c* and *S*. Therefore, we can accurately evaluate the probabilities of a particular volume even though no single glacier's volume in the distribution is known with great precision. Similarly, if a subset of glaciers is selected randomly from the set of all possible glaciers, then a regression of their volume and area data will give reasonable results, even though *c* may vary widely from glacier to glacier [*Farinotti and Huss*, 2013].

Likewise, by the law of large numbers, the sum of many glacier volumes will give a reasonable approximation of their total volume even if the errors in *c* are significant. In this case, the mean value of *c* is a reasonable choice for all glaciers in the summation [e.g., *Radić and Hock*, 2011; *Radić and Hock*, 2010; *Meier et al*., 2007], although with no loss of accuracy, the aggregate volume of a set of glaciers could be determined if values of *c,* selected in a random unbiased manner from the distribution of available values, were assigned to each glacier individually. The total sea‐level equivalent mass of all the world's glaciers can be determined with great precision from volume‐area scaling because the law of large numbers guarantees that the sum of hundreds of thousands of glaciers will approach the exact total volume very closely. The more glaciers that are included in the summation, then the more precise the result [*Farinotti and Huss*, 2013].

The scaling parameter *c* can be eliminated altogether when estimating global (or regional) *changes* in aggregate ice volume [e.g., *Oerlemans et al*., 2007; *Bahr et al*., 2009; *Leclercq et al*., 2011]. Following the derivation of *Bahr et al*. [1997], the total global (or regional) ice volume *V_T_* is estimated from each glacier's current volume *V* before volume changes are projected into the future. In particular, from the strong law of large numbers,

(144) $$V_{T}=\sum_{N}\;V=NE\left(V\right)$$

where *E* is the expected (mean) value and *N* is the total number of glaciers. Similarly, the expected (mean) change in a glacier's volume is

(145) $$E\left(p_{V}V\right)=E\left(p_{V}\right)E\left(V\right)$$

where *p_v_* is the fractional change in the glacier's volume—a ratio of the change in volume to the original volume. This transformation from the “mean of a product” to the “product of a mean” assumes that *p_v_* and *V* are independent; in other words, today's glacier knows nothing about tomorrow's change in climate. The change in total global ice volume Δ*V_T_* can then be estimated as

(146) $$\Delta V_{T}=\sum_{N}\;p_{v}V=NE\left(p_{v}V\right)=NE\left(p_{v}\right)E\left(V\right)=E\left(p_{v}\right)V_{T}$$

In this way, volume errors are restricted to the estimate of the mean fractional change *E*(*p_v_*). The fractional change *p_v_* is a ratio of volumes which eliminates the scaling parameter *c* and therefore minimizes errors.

*Möller and Schneider* [2010] propose an interesting approach that uses scaling on a single glacier at multiple points in time. This creates an ensemble of data for one glacier, similar to approaches above which consider ensembles of data collected from many different glaciers. They then demonstrate that the variability in *c* gives a reasonable regression with a fixed scaling exponent. We disagree with their statement that *c* = 1 for a glacier in equilibrium because this is not supported by the above theory (see equation (121)), but their general approach is a promising way to consider scaling on single glaciers.

The statistical accuracy of total volume estimations derived from volume‐area scaling has been evaluated in detail by *Farinotti and Huss* [2013]. Their analysis is an important step toward understanding both the limitations of scaling as a practical tool and the number of glaciers needed for a sufficiently large population. However, their specific methodology needs to be improved by limiting *γ* to a fixed value of 1.375 (glaciers) or 1.25 (ice caps). Their experiments allow *γ* to vary in time and/or space by regressing subsets of the synthetic data (see their experimental steps A.3, B.3, and C.3). Using regressions to determine an appropriate value for *γ* cannot be recommended (see section 8.2) and could lead to errors in their analysis.

### 8.6 Volume‐Area Scaling Cannot Be Applied to Fractional Parts of a Glacier

While volume‐area scaling is best applied to large sets of glaciers and can be applied to individual glaciers only with caution and considerable uncertainty, it can be applied to separate parts of a glacier only under specific limited conditions [*Bahr*, 2011]. An arbitrary subdivision of a single dynamically connected ice body will significantly underestimate the correct volume.

Consider, for example, a glacier of size *S* that is divided into two separate parts or branches, each with half of the total area. The correct total volume is *V* = *cS* ^*γ*^. However, each of the separate parts with area *S*/2 would be predicted to have partial volume

(147) $$V_{\text{part}1}=V_{\text{part}2}=c{\left(\frac{S}{2}\right)}^{\gamma }={\left(\frac{1}{2}\right)}^{\gamma }cS^{\gamma }$$

The sum of the volumes for each part should equal the total volume *V*, but

(148) $$V_{\text{part}1}+V_{\text{part}2}=2\;{\left(\frac{1}{2}\right)}^{\gamma }cS^{\gamma }=2{\left(\frac{1}{2}\right)}^{\gamma }V\neq V$$

Clearly, the correct total volume *V* is not equal to the sum of the parts, and for *γ* = 1.375, the estimated summed volume is only 77% of the correct volume *V*. Dividing a glacier into different numbers of fractional parts of unequal sizes can produce errors as large as 100% of the correct value.

It is common flow modeling practice to divide a single large ice mass into multiple glaciers separated along flow divides. If a glacier complex with separate termini is subdivided only along boundaries across which no ice flows, those subregions can be considered separate entities to which volume‐area scaling can be applied individually. Expressed more informally, if the boundaries isolating some subregion from adjacent ice could be reasonably replaced by a bedrock margin with no significant changes to the flow dynamics of that subregion, then each subregion is a separate entity (rather than a fraction of a whole glacier). In this case, volume‐area scaling will give a reasonable approximation to each separate subregion. One example of this situation is two glaciers that meet at their head along a buried arête that is only barely under the ice surface; each glacier is then dynamically and geometrically separate. In contrast, if the two glaciers meet at a flow divide with deep ice over the ice divide, then the ice mass is a single ice cap rather than two separate glaciers. This difference is more than semantic: an ice cap has different closure conditions and a different volume‐area scaling exponent. The choice of an appropriate volume‐area scaling exponent (ice cap versus glacier) needs to be evaluated on a case‐by‐case basis.

Some analyses have improperly treated glacier complexes as single glaciers [e.g., *Grinsted*, 2012; *Raper and Braithwaite*, 2006]. However, there is a similar danger in always treating an ice mass as a collection of separate glaciers without consideration of the depth of the ice along the flow divide [*Radić and Hock*, 2014; *Radić and Hock*, 2010; *Meier et al*., 2005; *Clarke et al*., 2013]. An explicit numerical approach is probably a better tactic for single glaciers [e.g., *Huss and Farinotti*, 2012] and highlights an important advantage of numerical models and a significant disadvantage of scaling in certain applications.

### 8.7 Derivatives of the Volume‐Area Scaling Relationship Are Valid Only for Infinitesimal Changes in Volume

Scaling is a natural choice for estimating future changes in area and volume. For a change in area Δ*S*, the final change in glacier volume Δ*V* can be estimated as

(149) $$V+\Delta V=c\;{\left(S+\Delta S\right)}^{\gamma }$$

Rearranging to put the volume *V* on the right‐hand side,

(150) $$\Delta V=c\;{\left(S+\Delta S\right)}^{\gamma }-c\;S^{\gamma }$$

This relationship is valid for any change in glacier area and volume, both large and small. Examples that treat changes in volume as finite quantities with a finite response time include *Marzeion et al*. [2012] and *Bahr et al*. [2009]. Note that many publications calculate changes in volume as the difference between an initial and final volume where each are estimated from volume‐area scaling [e.g., *Radić and Hock*, 2011; *Radić and Hock*, 2010; *Van de Wal and Wild*, 2001; *Slangen and Van de Wal*, 2011]. This approach is equivalent to the finite differencing above (150).

We can also consider infinitesimal changes in volume. Dividing (150) by Δ*S* and taking the limit as Δ*S* approaches zero

(151) $$\lim_{\Delta S\to 0}\frac{\Delta V}{\Delta S}=\lim_{\Delta S\to 0}\frac{c\;{\left(S+\Delta S\right)}^{\gamma }-c\;S^{\gamma }}{\Delta S}$$

This limit reduces to the derivative of the volume‐area scaling relationship,

(152) $$\frac{dV}{dS}=\;\gamma cS^{\gamma -1}$$

Rearranging gives the approximation

(153) $$dV=\;\gamma cS^{\gamma -1}dS$$

Equation (153) appears to represent a change in volume for a given change in area, but it has been derived strictly for infinitesimal changes in area. The derivative of the volume‐area scaling relationship (152) is inaccurate for most finite and large changes in area like those frequently observed and predicted in climate change scenarios [cf. *Harrison et al*., 2001; *Adhikari and Marshall*, 2012]. Finite volume changes must be calculated as shown in (150).

### 8.8 Testing the Volume and Area Scaling Relationship

Glacier surface area can be measured with field surveys, aerial images, or from satellite images as in much of the Randolph Inventory [*Pfeffer and the Randolph Consortium*, 2014]. Volume can be measured as average measured thickness times measured surface area (length times width times height). The theoretical volume‐area scaling relationship can be tested with regressions of independent, empirically derived volume‐area data, though power law regressions can be more complicated than an ordinary least squares approach that might weight large glaciers too heavily. An appropriate power law regression framework exists [e.g., *Clauset et al*., 2009] and has been used in glaciological contexts by *Grinsted* [2013], *Bahr and Radić* [2012], and others.

The vast majority of volume measurements come from radio echo sounding and borehole measurements combined with information about the surface area. As discussed by *Haeberli et al*. [2007] this might lead to an invalid regression of surface area against itself that introduces an artificial correlation. An alternative might be a regression of average thickness versus surface area (essentially *V*/*S* versus *S*), but available thickness data cover less than 2 orders of magnitude (Figure 2) which is insufficient to ensure that any hypothesized trend (power law or otherwise) rises above the noise. This limits the utility of thickness‐area regressions as a test of the scaling theory, but regressions of other scaling relationships might be used to test the general scaling theory instead: for example, scaling relations constructed between any two continuum parameters using (138).

From the mean value theorem of calculus,

(154) $$V=\int_{S}h\;dS=\bar{h}\;S$$

where $\bar{h}$ is defined as the average glacier thickness. This illustrates the necessary association between volume and area when integrated from thickness. The integral strictly applies only in the ideal case of thickness measurements that continuously cover the entire glacier area. In practice, however, thickness measurements are sparse and generally available only at uneven intervals. Thickness values for the rest of the glacier are estimated by interpolation and extrapolation from the measurements.

This practical distinction between continuous and sparse thickness data means that volume estimates derived from radio‐echo soundings may not contain a measure of the total surface area. Let Δ*S*_*i*_ be the section of the surface area associated with the thickness value *h_i_*. The Δ*S*_*i*_ do not have to be of uniform size. For *n* measured, interpolated or extrapolated values of the thickness, the volume is approximated as

(155) $$V=\int_{S}h\;dS\approx \sum_{i=1}^{n}h_{i}\;\Delta S_{i}$$

subject to the constraint that

(156) $$S=\sum_{i=1}^{n}\Delta S_{i}$$

For unevenly spaced data (unequal Δ*S*_*i*_), the total surface area (156) cannot be factored from the volume (155) except under improbable conditions such as a constant glacier thickness. The volume is thus not being approximated as a product of the surface area and average thickness. The techniques used by any particular glacier volume estimate would need to be addressed on a case‐by‐case basis, but it is not necessary to disregard all volume measurements as recommended by *Haeberli et al*. [2007].

Nevertheless, caution is warranted. If the thickness data are interpolated and extrapolated evenly across the entire glacier so that Δ*S*_*i*_ = Δ*S* for all *i*, then

(157) $$\begin{array}{c} V\approx \sum_{i=1}^{n}h_{i}\;\Delta S_{i}\\ =\Delta S\sum_{i=1}^{n}h_{i}\;\\ =n\;\Delta S\left(\frac{1}{n}\sum_{i=1}^{n}h_{i}\;\right)\\ =S\bar{h} \end{array}$$

Therefore, if a specific choice was made to use evenly spaced interpolations, then the volume estimate will contain a measure of the surface area, and an artificial correlation between area and volume (determined as in (157)) may occur.

It is very important to note, however, that despite occasional claims, volume as a theoretical concept does not *inherently* contain area nor does volume‐area scaling intrinsically relate area to itself on both sides of the equation [*Haeberli et al*., 2007; *Haeberli and Linsbauer*, 2013]. This misunderstanding has been discussed and refuted by *Lüthi et al*. [2008] and rejected elsewhere as well [e.g., *Raper and Braithwaite*, 2009]. As this review's derivations make clear, volume and area are theoretically independent continuum variables that (with some effort) can be measured independently. (For example, in theory, the volume is related to the glacier's mass via the density—we would not suggest that measuring the mass is a practical way to establish a glacier's volume, but it illustrates that volume is theoretically independent of the surface area.) The difficulty of measuring volume makes a test of the scaling theory more challenging, but similar obstacles have been overcome in other geophysical disciplines. For example, Hack's law relates river basin area to main channel length, and there is an extensive body of literature that theoretically derives, measures, and validates this important scaling relationship even though basin length might be interpreted (incorrectly) as appearing on both sides of the relationship [e.g., *Hack*, 1957; *Birnir*, 2008].

### 8.9 Numerical Models With Different Closure Conditions Will Predict Different Scaling Exponents

Using ensembles of glaciers, *Adhikari and Marshall* [2012] test a wide variety of closure conditions with a three‐dimensional Stokes model. Their results nicely illustrate the influence that changes in closure conditions can have on the volume‐area scaling exponent. *Radić et al*. [2007] perform similar experiments and find comparable results with a one‐dimensional flow line model.

Observations support a particular mass balance closure (*m* = 2), a particular width closure (*q* = 3/5), and a particular equilibrium AAR closure (AAR = 0.577); see equation (126). None of these are likely to be the exact values associated with any *single* glacier, but when considering ensembles of glaciers (see section 8.5), these closure conditions should hold on average. Therefore, before comparing the theoretically derived volume‐area exponent to numerically derived exponents, the numerical model should be tested to ensure at least one of the closure conditions holds on average and is consistent with the theory. If the closure conditions are not the same as those used in the preceding theory, then there is no a priori reason to expect that the numerical model will derive the same scaling exponent as the theory.

For example, in *Farinotti and Huss* [2013] the closure conditions may not be the same as those used to derive the theoretical volume‐area scaling exponent. In particular, the model specifies a mass balance gradient that (once the glacier is in equilibrium) may or may not be consistent with equations (126) and (127). This could potentially explain why their analysis finds scaling exponents that are slightly lower than the theoretical value. The potential impact of this difference on their analysis of scaling accuracy is unknown and not necessarily important or large but should be evaluated.

### 8.10 Volume‐Area Scaling Can Be Used to Test Numerical Model Performance

Valid closed form solutions are an excellent and essential test of a numerical model's general health. For example, glaciologists would be unlikely to trust a numerical model that cannot reproduce the analytical plane‐strain laminar‐flow solution under appropriate boundary conditions (uniform thickness, inclined plane, infinite length, width, etc.). Similarly, like other closed form solutions, power law scaling can confirm that a model is behaving appropriately. If the model uses the same closure conditions as the theory, then the model should predict the same exponents as the scaling theory. As an example, *Pfeffer et al*. [1998] used volume‐area scaling as a test of model performance on circular ice caps. With very few closed form solutions for glacier flow, this aspect of power law scaling relationships is underutilized.

### 8.11 Numerical Models Are Not Inherently More Accurate Than Volume‐Area Scaling

Numerical models can incorporate complicated real‐world geometries, while volume‐area scaling does not have the capacity to explicitly include ice falls, nunataks, or any of the other myriad geometric complexities found in an actual glacier. It would seem obvious that numerical models can offer a more refined estimate of an individual glacier's volume. However, without information about the basal boundary (e.g., thickness and basal slip), numerical models typically derive their results solely from surface data (surface geometry, surface velocities, surface mass balance, etc.). This unbalanced placement of the boundary conditions leads to an ill‐posed inversion with an inherent instability that amplifies short spatial wavelengths and limits model predictions to long spatial wavelengths [e.g., *Bahr et al*., 2014; *Raymond and Gudmundsson*, 2005; *Zhdanov*, 2002; *Bahr et al*., 1994; *Balise and Raymond*, 1985; *Lliboutry*, 1987, p. 178]. This is a theoretical limitation, and the instability cannot be removed by choosing a different numerical model, a more accurate model, or even an analytical model. As a consequence, *Bahr et al*. [2014] show that any estimate of volume (whether numerical or analytical) must be limited to low spatial resolutions, and these resolutions are similar for both numerical models and for volume‐area scaling. When all else is equal, the errors created by the ill‐posed inversion put volume‐area scaling and numerical models on an equal footing for volume approximations.

For entirely different reasons, volume‐area scaling is best applied to ensembles of glaciers and can be inaccurate when applied to individual glaciers (see section 8.5). The scaling parameter *c* has a distribution of possible values, and unless the value of *c* is well‐established for the particular glacier under consideration, then a numerical inversion model like *Huss and Farinotti* [2012] or *Clarke et al*. [2013] would be an excellent choice for calculating an individual glacier's volume. However, if any of the numerical models' parameters are unknown or have a distribution of possible values (e.g., tunable sliding law parameters, flow law rate parameter, shape factors, and continentality factors), then the relative accuracy of the numerically modeled volume versus the scaled volume would have to be assessed for that particular model. Choosing (or tuning) reasonable values for the unknown numerical model parameters is no different than choosing (or tuning) reasonable values for the unknown scaling parameter *c*, and there is no a priori reason to expect that a tuned model will be more accurate than a tuned scaling solution.

### 8.12 Complex Glacier Geometries Are Well Represented by Scaling Relationships

Dimensional analysis and scaling have been used to test and characterize complex models of cratering, explosions, and turbulence [e.g., *Schmidt and Housen*, 1995; *Welty et al*., 1984]. Despite some conjectural statements in the glaciological literature [e.g., *Huss and Farinotti*, 2012; *Grinsted*, 2013], there is no observational or theoretical consideration that would suggest large or multibranched glaciers will be more poorly represented by scaling laws than these other complex physical phenomena. In fact, some theoretical considerations suggest that additional (rather than fewer) power laws may be discovered for the most complex dendritic glacier geometries [*Bahr*, 2009]. Complex geometries may in fact be especially well suited to power laws.

Power laws are routinely used to represent water flowing in river basins that are substantially more dendritic than glaciers, with tens of thousands of branching stream tributaries. Hack's law, for example, scales total basin drainage area to main channel length [*Hack*, 1957] and applies just as accurately to a small brook as to the complex Mississippi River system [e.g., *Peckham and Gupta*, 1999]. Given the significant similarities between river valleys and glacier valleys, as well as the notable similarities between flowing ice and water, there does not appear to be any reason to expect volume‐area scaling or other power laws to fail for glaciers because of either size or complexity.

### 8.13 Individual Glacier Volume Is Not Uniquely Defined by Scaling

Given the surface area of an individual glacier, the corresponding individual volume is *not* uniquely defined. This is true in both theory and reality. For example, consider all of the world's glaciers that have a certain specified area within plus or minus some arbitrarily small tolerance. With great certainty, we know that these glaciers will not each have the same volume because they are each on different basal topographies in different local climates. For the particular set of glaciers with this specified area (plus or minus the tolerance), there will be a distribution of associated glacier volumes. The distribution of volumes may be small and may have a well‐defined mean; but the volume cannot and intuitively should not be uniquely determined from the area.

This is in agreement with theory. At first glance, volume‐area scaling may look like a bijective relationship (one‐to‐one correspondence between volume and area), but *c* is not unique. The scaling parameter *c* has a distribution [*Bahr*, 1997a] that depends on the values associated with certain dimensionless parameters that can vary from glacier to glacier (equations (121) and (134)). Therefore, given an area, there will be a distribution of possible volumes associated with the distribution of values for *c*. Because the volume is not uniquely defined, volume is best described as a probabilistic distribution for ensembles of many glaciers. For example, by the law of large numbers, the sum of many scaled volumes will closely approximate (to high order) the actual total volume, as described in section 8.5.

In addition, power law scaling describes a relationship between two characteristic values. These characteristic values (as described in section 6.2) can be selected in many different ways, and the choice only needs to be consistent from glacier to glacier. For example, we could choose the characteristic area of a glacier to be the accumulation area, the cross‐sectional area at the equilibrium line, or the total area of the glacier. Some choices may not give a particularly useful measure of glacier volume, but scaling theory ensures that the resulting characteristic volume will be internally self‐consistent for all glaciers. In this sense as well, volume‐area scaling is not a unique relationship. Instead, volume‐area scaling describes a set of many possible relationships between many possible characteristic values. This is the same for all other scaling relationships (138). As a result, each scaling relationship is a powerful and general tool that can be applied to many different and seemingly unrelated applications (see section 6 for examples).

## 9 Future Directions

### 9.1 Extensions to Volume‐Area Scaling

Volume‐area is popular because its application is relatively easy and because area data are readily available while volume data are not. Nevertheless, in an attempt to better understand potential departures from the standard scaling relationship (1), a number of applications have considered more complex extensions that include slope and other geometric shape factors [e.g., *Grinsted*, 2013; *Adhikari and Marshall*, 2012]. Many of these assume that the scaling exponent *γ* will vary with these extensions. Instead, we suggest that future studies might wish to explore more rigorous theoretical extensions to volume‐area scaling or to derive and explore different scaling relationships altogether. Volume‐area scaling is by no means the only scaling relationship involving glacier volume (see equation (138)), and other relevant scaling relationships can be derived using the general dimensional techniques described above.

For example, if we are interested in potential variations due to average glacier slope, then equations (47) and (121) can be combined to show that

(158) $$c={c_{q}}^{\frac{-\left(m+1\right)}{\left(n+2\right)\left(q+1\right)}}{\left(\frac{\Pi_{4}\Pi_{9}c_{m}}{\Pi_{2}{\Pi_{6}}^{n}A\rho^{n}{g_{z}}^{n}}\right)}^{\frac{1}{n+2}}{\theta_{xz}}^{\frac{-n}{n+2}}$$

The scaling parameter *c* evidently contains information about the slope *θ_xz_*. Combining (158) with volume‐area scaling gives a new scaling relationship,

(159) $$V={c_{q}}^{\frac{-\left(m+1\right)}{\left(n+2\right)\left(q+1\right)}}{\left(\frac{\Pi_{4}\Pi_{9}c_{m}}{\Pi_{2}{\Pi_{6}}^{n}A\rho^{n}{g_{z}}^{n}}\right)}^{\frac{1}{n+2}}S^{\gamma }{\theta_{xz}}^{\frac{-n}{n+2}}$$

This extension does not invalidate volume‐area scaling or modify the original volume‐area scaling exponent, but simply makes the role of surface slope explicit. In practice, the slope term will cover little more than an order of magnitude, while the area term can cover 5 or more orders of magnitude. This makes the effect of slope secondary to surface area, but its inclusion in a three‐dimensional plot (volume versus area versus slope) might help to explain some of the variability otherwise attributed to *c*.

Other extensions to the scaling theory could include almost any glaciological parameter by using equations (136) and (138) or by using a different choice of characteristic value. For example, we could use the total vertical range *R* rather than thickness as the characteristic value for *z*. In that case, using *θ*_*xz*_ = *R*/*L* as a reasonable approximation of the surface slope, equations (159) and (119) expand to

(160) $$\begin{array}{c} V={c_{q}}^{\frac{-\left(m+1\right)}{\left(n+2\right)\left(q+1\right)}}{\left(\frac{\Pi_{4}\Pi_{9}c_{m}}{\Pi_{2}{\Pi_{6}}^{n}A\rho^{n}{g_{z}}^{n}}\right)}^{\frac{1}{n+2}}S^{\gamma }{\left(\frac{R}{L}\right)}^{\frac{-n}{n+2}}\\ ={c_{q}}^{\frac{-\left(m+1\right)}{\left(n+2\right)\left(q+1\right)}}{\left(\frac{\Pi_{4}\Pi_{9}c_{m}}{\Pi_{2}{\Pi_{6}}^{n}A\rho^{n}{g_{z}}^{n}}\right)}^{\frac{1}{n+2}}S^{\gamma }R^{\frac{-n}{n+2}}{\left(\frac{S}{c_{q}}\right)}^{\frac{n}{\left(n+2\right)\left(q+1\right)}}\\ ={c_{q}}^{\frac{-\left(m+1\right)-n}{\left(n+2\right)\left(q+1\right)}}{\left(\frac{\Pi_{4}\Pi_{9}c_{m}}{\Pi_{2}{\Pi_{6}}^{n}A\rho^{n}{g_{z}}^{n}}\right)}^{\frac{1}{n+2}}S^{\gamma +\frac{n}{\left(n+2\right)\left(q+1\right)}}R^{\frac{-n}{n+2}} \end{array}$$

These theoretically derived versions of volume‐area‐range and volume‐area‐slope scaling can be compared to proposed scaling relationships in *Grinsted* [2013], *Adhikari and Marshall* [2012], *Raper and Braithwaite* [2006, 2009], and elsewhere.

Combining existing dimensional parameters and existing scaling relationships is relatively straightforward. For this reason, when observations suggest a particular extension of the scaling theory, we urge similar theoretical derivations from the physics. As with all the scaling derivations, the exponents will generally be fixed by the physics, and the derived exponents can provide reasonable bounds for observed exponents.

### 9.2 Other Potential Directions

At its core, volume‐area scaling is a probabilistic relationship (see section 8.13) and could benefit from the stochastic approach used in modern statistical physics. To date, this connection has not been explored, but statistical physics has many useful techniques for handling ensembles of objects like collections of glaciers. The techniques of statistical physics could lead to a deeper understanding of scaling, just as it has for glacier calving laws [*Bassis*, 2011], phase transitions, fluid mechanics, and many other applications [*Stanley*, 1999].

The probabilistic nature of *c* is particularly troublesome for practical applications. Instead of choosing a single and poorly established value for *c*, we suggest that the explicit form of equations (121) and (134) be exploited to understand the theoretical range of possible values. In practice, the dimensionless parameters Π_2_, Π_4_, Π_6_, and Π_9_ have a limited range. Gravity and density have established values; the ratios of length to thickness are limited by the finite size of glaciers; and the ratios of horizontal to vertical velocities are also limited. This places constraints on the dimensionless parameters that could be used to constrain values of *c* in future studies.

The necessity of closure conditions is common in other disciplines. For example, statistical physics often fixes one scaling exponent to establish others (e.g., in phase transitions) [*Stanley*, 1999]. This is the same approach taken in equation (138) where all scaling exponents are written in terms of *γ* [*Bahr*, 1997a]. However, renormalization techniques in statistical physics have been used to establish exponents without reference to closure conditions. Future developments might explore similar methods to eliminate the closure condition in volume‐area scaling and other glacier scaling relationships.

Time dependence is poorly understood and rarely exploited in volume‐area scaling. Many if not most analyses incorrectly state or assume that volume‐area scaling is an equilibrium theory. The sea level application by *Marzeion et al*. [2012] is a good first step, as is the closely related theoretical work of *Lüthi* [2009], *Raper and Braithwaite* [2009], and *Harrison* [2013], and the three‐dimensional modeling experiments of *Pfeffer et al*. [1998] and *Adhikari and Marshall* [2012]. The use of response time scaling to model transient volume‐area behavior should be pursued in future studies. Exploring the temporal behavior of *c* might also lead to a deeper understanding of transient volume‐area behavior.

The distinction between ice caps and glaciers plays a pivotal role in scaling applications. The scaling exponents differ dramatically, and estimates of aggregate glacier volume and potential sea level rise can change significantly depending on the fraction of the world's ice masses that are defined as ice caps [e.g., *Radić and hock*, 2011; *Mernild et al*., 2013]. Better data are needed to delineate the world's glaciers from ice caps (this is not in the recent Randolph Inventory [*Pfeffer and the Randolph Consortium*, 2014]), and the theoretical differences need a close examination. As discussed in *Bahr* [2009], the physical distinction between ice caps and glaciers appears to be described by a phase transition in complexity which could be explored with techniques of statistical physics. Regardless of the theoretical approach, more accurate distinctions will improve our understanding of scaling and have immediate implications for sea level rise predictions.

## 10 Conclusions

Volume‐area scaling and related power laws have been derived by two different methods. No simplifications are necessary in either approach. The derivations do not assume plane strain, shallow ice, or steady state conditions. A single closure condition is required, and available data for three possible choices of the closure condition all give the same result. Each of these closure conditions imply the others and are self‐consistent.

Of the two scaling derivations, the dimensional analysis is the most fundamental. The dimensional analysis shows that glaciological scaling relationships are inherent, ubiquitous, and inevitable. As a consequence, volume‐area and other scaling relationships are not tied to any particular set of partial differential equations. No matter what equations are used to describe glacier flow, the volume‐area relationship must be valid, or we would violate the Buckingham Pi Theorem.

The alternative stretching symmetry analysis shows that volume‐area (and other) scaling can be derived from the constitutive, force balance, and continuity equations. These specific equations are at the foundation of continuum mechanics in glaciology, and it makes the physical origins of the scaling relationships clear. Each dimensionless parameter is tied to a particular equation or combination of equations.

The unabridged derivation of volume‐area scaling presented here should help to eliminate some of the most common misunderstandings and misapplications of the theory. Notably, the scaling exponent is a constant, and any deviation from the theoretical value requires changes to the closure conditions. This is possible, but unlikely and would need careful justification. Furthermore, the scaling exponent is not a function of time and making it a function of time leads to inherent contradictions. Instead, the scaling parameter *c* should be treated as a variable that can change with time and as a function of several dimensionless parameters. The choice of *c* can and will change from glacier to glacier.

For consistency with the underlying theory, we suggest the following guidelines for practical applications of volume‐area scaling. Because of the theoretical connection (138) between all scaling relationships that use fundamental continuum variables, nearly identical guidelines will apply to other glacier scaling relationships as well.

1. Fix the scaling exponent to the theoretical constant *γ* = 1.375. Let the multiplicative scaling parameter *c* vary as necessary; this variability could be by glacier, by region, by time, by slope, by climate parameters, or by many other possible factors.
2. Apply volume‐area scaling to *collections* of many glaciers, not to individuals. Treat the resulting set of volumes as a probability distribution. Alternatively, sum the predicted volumes of many individual glaciers for an accurate estimate of the aggregate volume.
3. Volume‐area scaling should only be applied to an individual glacier if the result is treated as an order of magnitude estimate.
4. Include time dependence by using response time scaling and by considering the time dependence of parameters that define *c* in equations (121) and (134). Do not let *γ* vary with time because this leads to theoretical contradictions.
5. Avoid applying volume‐area scaling to glacier complexes. Do not apply volume‐area scaling to individual parts or branches of a glacier.
6. Compare scaled volumes to numerically modeled volumes if and only if the model is using the same closure conditions.
7. If another parameter like slope or elevation range is relevant to an application, then extensions of the theory and new scaling relationships (like volume‐area‐slope) may provide answers (e.g., equations (159) and (160)). Beyond the well‐known volume‐area and response time scaling relationships, the potential of scaling theory as a tool for glaciology has only barely been exploited and explored.
